# The Potential Distribution of Invading *Helicoverpa armigera* in North America: Is It Just a Matter of Time?

**DOI:** 10.1371/journal.pone.0119618

**Published:** 2015-03-18

**Authors:** Darren J. Kriticos, Noboru Ota, William D. Hutchison, Jason Beddow, Tom Walsh, Wee Tek Tay, Daniel M. Borchert, Silvana V. Paula-Moreas, Cecília Czepak, Myron P. Zalucki

**Affiliations:** 1 CSIRO, GPO Box 1700, Canberra, ACT, Australia; 2 School of Biological Sciences, Faculty of Science, The University of Queensland, Queensland, 4072 Australia; 3 CSIRO, Private Bag 5, Wembley WA, Australia; 4 Department of Entomology, University of Minnesota, St. Paul, Minnesota, United States of America; 5 Department of Applied Economics, University of Minnesota, St. Paul, Minnesota, United States of America; 6 Animal and Plant Health Inspection Service-Plant Protection and Quarantine-Center for Plant Health Science and Technology, Plant Epidemiology and Risk Analysis Laboratory, Raleigh, North Carolina, United States of America; 7 Embrapa Cerrados, BR 040 km 18, Planaltina-DF, Brasil; 8 Escola de Agronomia e Engenharia de Alimentos, Universidade Federal de Goiás. Campus II, Caixa Postal 131, CEP, Goiânia, Brasil; Institute of Plant Physiology and Ecology, CHINA

## Abstract

*Helicoverpa armigera* has recently invaded South and Central America, and appears to be spreading rapidly. We update a previously developed potential distribution model to highlight the global invasion threat, with emphasis on the risks to the United States. The continued range expansion of *H*. *armigera* in Central America is likely to change the invasion threat it poses to North America qualitatively, making natural dispersal from either the Caribbean islands or Mexico feasible. To characterise the threat posed by *H*. *armigera*, we collated the value of the major host crops in the United States growing within its modelled potential range, including that area where it could expand its range during favourable seasons. We found that the annual value of crops that would be exposed to *H*. *armigera* totalled approximately US$78 billion p.a., with US$843 million p.a. worth growing in climates that are optimal for the pest. Elsewhere, *H*. *armigera* has developed broad-spectrum pesticide resistance; meaning that if it invades the United States, protecting these crops from significant production impacts could be challenging. It may be cost-effective to undertake pre-emptive biosecurity activities such as slowing the spread of *H*. *armigera* throughout the Americas, improving the system for detecting *H*. *armigera*, and methods for rapid identification, especially distinguishing between *H*. *armigera*, *H*. *zea* and potential *H*. *armigera* x *H*. *zea* hybrids. Developing biological control programs, especially using inundative techniques with entomopathogens and parasitoids could slow the spread of *H*. *armigera*, and reduce selective pressure for pesticide resistance. The rapid spread of *H*. *armigera* through South America into Central America suggests that its spread into North America is a matter of time. The likely natural dispersal routes preclude aggressive incursion responses, emphasizing the value of preparatory communication with agricultural producers in areas suitable for invasion by *H*. *armigera*.

## Introduction

*Helicoverpa armigera* (Hübner) (Lepidoptera; Noctuidae) has recently extended its already considerable geographical range from Europe, Africa, Asia and Australasia to the New World. It was formally reported as present in Brazil [1,2,3,4,5,6] and Paraguay [7] in 2013, and Argentina in 2014 [8], but given the extent of the area infested and high abundance (see below), it is likely to have been present in South America for some time before detection. Most recently it has been reported in Bolivia, Uruguay (Cecilia Czepak, Universidade Federal de Goias Brazil, pers. comm.) and Puerto Rico [9]. Naturally, biosecurity managers and others in the Americas who may be impacted by the spread of *H*. *armigera* are eager to understand the potential geographical range and abundance of this notorious pest species better.

*Helicoverpa armigera* is a polyphagous pest of agricultural crops. We are aware of two pest risk assessments for *H*. *armigera*; for the USA [10] and for Europe [11]. The risk assessment for the USA focused attention on the *H*. *armigera* incursion risks posed by the movement of passengers and goods. In contrast, the European assessment acknowledged the importance of natural dispersal of *H*. *armigera* in the risk profile in Europe, and consequently the lack of options for managing the recurrent incursion risks in Northern Europe [11].

Using a CLIMEX model, Zalucki and Furlong [12] indicated broadly where in the Americas *H*. *armigera* could establish successfully should it be introduced. Here we refine and extend that model to alert biosecurity managers to the potential for invasion, identifying areas that are suitable for establishment and those that are suitable for population growth during favourable seasons. We consider where host crops are grown and the effects of irrigation in extending the range of this species. In addition, we examine border interception data for this species as a means of contextualizing the invasion risks. Our objective is to detail areas at risk in North and South America at a fine spatial scale, discuss the risk of spread and impacts, and suggest interim mitigation strategies to delay the apparently inevitable invasion of North America.

## Methods

### Helicoverpa armigera: Background Biology and Ecology

Generally *H*. *armigera* is referred to as a single species in the majority of literature, but there are reports of three subspecies around the world, and no evidence of reproductive isolation, at least between the two most widespread subspecies of *H*. *a*. *conferta* (present in Australia, and possibly New Zealand) and *H*. *a*. *armigera* (found in the rest of the Old World) [13,14]. Given that we do not know the geographical origin of *H*. *armigera* that recently invaded the New World, it is logical to follow the convention of a single species with a wide potential range.

**Fig 1:**
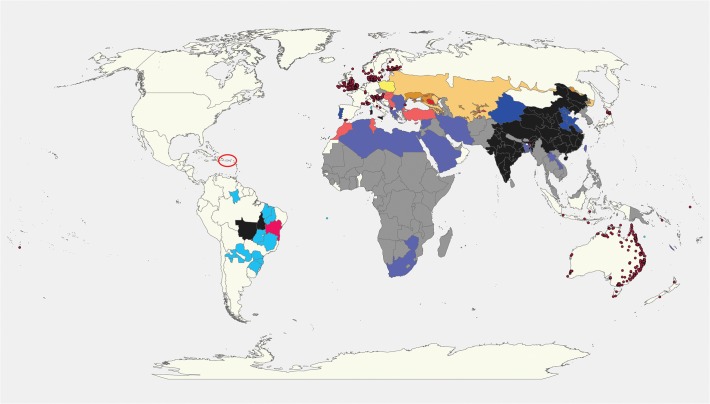
The known global distribution of *Helicoverpa armigera*. Source GBIF and Matthews [13] (point location records), AgroAtlas (http://www.agroatlas.ru/en/content/pests/Helicoverpa_armigera/map/) for sub-regional mapping, CABI and Alejo Costa (Syngenta, pers. comm.), Cecilia Czepak (Universidade Federal de Goiás, pers. comm.) and Miguel F. Soria (IMAmt, Mato Grosso Cotton Institute, pers. comm.) for country or state level records. Where point location records were available, coarser level records have been ignored. Categories such as ‘widespread’ should be interpreted cautiously. For example, in Northern Africa it may only be widespread throughout the very restricted cropping areas adjacent to the Mediterranean Sea or inland irrigation areas.

In the Old World, the species has been a major pest of agriculture, horticulture and floriculture throughout its range (Fig. 1). There is an extensive literature on the species, although it tends to be necessarily parochial, as researchers concentrate on managing the species in particular localities (e.g., [15,16,17]). Nevertheless some general statements can be made that are likely to be more widely applicable.

*Helicoverpa armigera* is polyphagous; females lay eggs, and the caterpillar stage can survive and feed on a very wide range of host plant species [18,19]. Not surprisingly, many of these hosts are crops, including many field crops: cotton, sorghum, sunflower, chickpeas, lucerne, lupins, soybeans, tobacco, maize and wheat; and horticultural crops such as tomatoes, lettuce, capsicum, various bean crops, and flowers: chrysanthemums, gladioli and roses. In Australia, 35 plant families have been recorded as hosts of the species [15,20]. Host use for *H*. *armigera* in countries outside Australia has received less attention, with the exception of India [21]. Published records for *H*. *armigera* host use in China [22], Europe [23,24] and Africa and the Middle East [25,26,27] do little more than catalogue its status as a pest of local crops. *Helicoverpa armigera* is recorded from 68 plant families worldwide, but only 14 families are recorded as containing a host in all geographical areas [19].

The pest status of *H*. *armigera* is in part a function of the plant parts eaten. Although larvae can feed and survive on leaves of many hosts [15], they move to [28,29,30], prefer to feed on [31], and do better in terms of survival, subsequent fecundity and other fitness parameters when feeding on flowers and fruit (e.g., [32,33]); hence the common names budworm, bollworm, earworm that belie its varied feeding habits. Interestingly one of its common names is the “American Bollworm,” due in part to confusion surrounding the *Helicoverpa*/*Heliothis* clade in the Heliothinae.

Females can lay up to 3 000 eggs under laboratory conditions but in general potential fecundity lies in the range 500–1 000 and depends on the rearing host and ambient conditions [34]. Realised fecundity is a function of adult longevity (generally 7–20 days in the laboratory (e.g., [33])), weather conditions and hosts availability [35], although there are no good estimates of realised fecundity under field conditions.

Larvae develop through 5–7 instars and their physiological thermal requirements have been estimated (e.g., [34,36,37]). Minimum developmental thresholds are *ca* 11–12°C, and it takes approximately 475 degree-days from egg to adult [34]. The optimum temperature for development is around 31–34°C, with an upper threshold of *ca* 37–42°C depending on what model is fitted to data, and whether temperatures were fluctuating or constant [34].

The level of damage caused in a crop depends in part on the abundance of adult moths, the number of eggs they lay, and the numbers of larvae surviving to the larger damaging larval instars. *Helicoverpa armigera* adults are migratory, and can move many hundreds of kilometres between regions and extensively between fields within regions [38,39,40,41]. Thus, influxes of moths may occur into an area from far away, or from nearby fields. Depending on the crop plant and season, complete crop loss can result if caterpillars are left unchecked [42].

Apart from the significant vagaries of migration [43], the level of *H*. *armigera* attack (pest pressure) that a particular agricultural producer faces in a particular season on a particular crop depends on the favourability of the region for the pest. This is determined by regional and on-farm cropping system factors, including cultivation practice, adjacent crops, percentage of alternative host crops/plants in the region, and so forth (e.g., [17,39,44]). Climate favourability within and between seasons can be a major determinant of abundance, as rainfall and temperature conditions impact on all life history stages [12,35,45,46].

In subtropical and temperate parts of its geographical range, *H*. *armigera* enters a facultative cold diapause in the pupal stage during the winter months [47]. In tropical areas the proportion of the population entering diapause is very small, and, rainfall and host plants permitting, populations breed year round [48]. Diapause has been studied in different parts of the species range, including: Africa [49,50], Australia [51,52,53], Greece [54], Israel [41], India [55], China [56] and Japan [57]. In general, declining photoperiod and temperatures experienced by the larval and pre-pupae stages determine the proportion of the population entering cold diapause, with winter/spring temperatures determining the timing of emergence. In China, Wu and Guo noted that the supercooling point for diapausing pupae (approximately −21°C) was considerably cooler for populations of *H*. *armigera* collected from more continental northwestern Chinese locations, than those from eastern China [58]. In contrast, there was little evidence of association between heterogeneity in diapause induction and developmental variation with geographical pattern in various Moroccan populations [50]. In China, the ability of *H*. *armigera* pupae to tolerate −16°C was reduced to negligible levels when soil moisture was increased from 20% to 80%. As befits its wide latitudinal range, *H*. *armigera* also experiences aestivation, with induction triggered by the larval temperature experience [59]. In combination, these two diapause mechanisms confer a large degree of protection to *H*. *armigera* from the extremes of temperature, albeit at the expense of some growth potential during marginally suitable climate.

### CLIMEX

CLIMEX is a bioclimatic niche model that has been demonstrated to be well-suited to estimating the potential distribution of a wide range of taxa [6,60]. The CLIMEX Compare Locations model [6,60] was used to estimate the climatic suitability for *H*. *armigera* globally, taking into account the effects of irrigation in extending the growing season in semi-arid areas, and the need for suitable crop hosts to be available. The climate dataset used here was the CliMond gridded 10’ spatial resolution historical dataset centred on 1975 (CM10_1975H_V1.2) [61]. This dataset consists of long-term monthly average values for minimum temperature, maximum temperature, precipitation, and relative humidity at 0900 and 1500 hr. The model-fitting strategy involved refitting the CLIMEX model for *H*. *armigera* developed by Zalucki and Furlong [12], fitting it to distribution records in Australia and Asia, and verifying it with distribution records elsewhere. The distribution records in Central Australia indicated in [12] and those in northern Europe include reports of transient populations. The northern limit for records of permanently established populations in Europe is in southern France, Bulgaria and northern Greece [11]. For the model-fitting exercise, areas with transient populations should have a positive Annual Growth Index (GI_A_), and an unsuitable Ecoclimatic Index (EI).

The CLIMEX parameter set of Zalucki and Furlong [12] was firstly modified to remove internal parameter inconsistencies where the lower (SM0) and upper (SM3) soil moisture growth limits overlapped thresholds for the accumulation of Dry Stress (HDS) and Wet Stress (HWS) respectively. To facilitate this, it was also necessary to reduce the upper optimum soil moisture level (SM2) and the upper soil moisture limit (SM3). As a result of the increase in the lower Soil Moisture threshold (SM0), the Dry Stress accumulation rate (HDS) was relaxed slightly.

CLIMEX can simulate three types of cold stress mechanism. Damaging low temperatures can destroy tissues, and tends to have a rapid stress accumulation rate. If the diurnal heat sum above the developmental base temperature is insufficient, an organism may be unable to generate or forage for sufficient energy resources to offset basal respiration losses, and their condition wanes slowly. For ground nesting animals that are able to regulate their temperature environment somewhat, daily average temperatures can limit the ability of a population to persist [62]. The Cold Stress mechanism in the CLIMEX model was changed from a damaging temperature mechanism to a degree day mechanism. In Australia, the apparent southern latitudinal limit for persistent populations was similar for both mechanisms, but the fit elsewhere throughout the poleward range was far better using the degree day model (e.g., in Western Australia). Morey *et al*. note that *H*. *zea* survives poorly when exposed to conditions close to freezing [63]. Whilst this may also be true of *H*. *armigera* larvae or adults, we found no need for this stress mechanism in order to fit the available distribution data, probably because the population can survive sub-freezing conditions through facultative pupal diapause.

The Heat Stress accumulation rate (HDS) was increased to fit the known inland distribution in Australia better. Compared with the model of Zalucki and Furlong [12], these modifications resulted in a significant reduction in the area in central Australia that was apparently suitable for supporting persistent populations. These semi-arid areas are now modelled as having a mildly positive Annual Growth Index (GI_A_), but an unsuitable Ecoclimatic Index, implying that these areas can support ephemeral populations during favourable seasons or years [20]. The Cold Stress modifications allowed the potential range to extend further southward and into Western Australia where persistent populations have been recorded. The modified parameter set is presented in Table 1.

**Table 1:** CLIMEX Compare Locations model parameters for *Helicoverpa armigera* (mnemonics are taken from [6,60]).

Index	Parameter	Previous Values	New Value^a^
Temperature	DV0 = lower threshold	11°C	11°C
	DV1 = lower optimum temperature	20°C	20°C
	DV2 = upper optimum temperature	31°C	31°C
	DV3 = upper threshold	37°C	37°C
Moisture	SM0 = lower soil moisture threshold	0.05	**0.1**
	SM1 = lower optimum soil moisture	0.7	0.7
	SM2 = upper optimum soil moisture	2.0	**1.0**
	SM3 = upper soil moisture threshold	4.0	**2.0**
Cold stress	TTCS = temperature threshold	9	-
	TTHS = stress accumulation rate	-0.0003	-
	DTCS = degree day threshold	-	**5°C days**
	DHCS = stress accumulation rate	-	**−0.0005 week^−1^**
Heat stress	TTHS = temperature threshold	37°C	37°C
	THHS = stress accumulation rate	0.0005 Week^−1^	**0.001 Week^−1^**
Dry stress	SMDS = soil moisture threshold	0.1	0.1
	HDS = stress accumulation rate	-0.005 Week^−1^	**−0.004 Week^−1^**
Wet Stress	SMWS = soil moisture threshold	2	2
	HWS = stress accumulation rate	0.005 Week^−1^	0.005 Week^−1^
Diapause Index	DPD0 = Diapause induction daylength	11 h	11 h
	DPT0 = Diapause induction temperature	15°C	**10°C**
	DPT1 = Diapause termination temperature	16°C	**10°C**
	DPD = minimum days in diapause	69	**0**
	DPSW = summer/winter switch	0 (winter)	0 (winter)

Model parameters were adapted from Zalucki and Furlong [12]. Changed values are indicated in bold.

^a^ Values without units are dimensionless indices of soil moisture for a 100 mm single bucket model (0 = oven dry, 1 = field capacity).

The sensitivity of the model parameters was investigated using a newly developed function in CLIMEX (Kriticos *et al*., in prep.). The function adjusts the parameter values up and down by a nominated range, and then reports the corresponding change in the state variables (Table A in S1 File).

#### Model verification

The fit of the model was compared with results from Zalucki and Furlong [12], taking into account the use of a station-based dataset in [12] and a gridded climate dataset here, and the expected minor idiosyncrasies between datasets. We plotted the known distribution of *H*. *armigera* based on the CABI 1993 map [64] and the more recent offering (2013) [65]. For Australia we used vouchered specimen collection records summarised in Matthews [13] to generate a distribution map, as was done in Zalucki and Furlong [12]. Lines and dots on maps can be misleading: species distribution and abundance tends to be dynamic, shifting or waxing and waning in both time and space [66]. That temporal variability in distribution is most evident in “marginal” areas such as Western Queensland and NSW (Fig. 1). Positive collection records for the species here are rare and only occur in those seasons and years that favour host plant growth [20].

Because CLIMEX models the weekly suitability of climate for population growth, it is possible to verify that aspect of the model as well as the geographical patterns of establishment. The modelled growth phenology (GI_W_) was compared with field data from Punjab (India), Xinjiang (China), and Northern New South Wales (NSW, Australia).

#### Diapause

The facultative diapause in *H*. *armigera* populations may be genotypically variable, with only a proportion of individuals entering diapause when conditions are suitable [48]. Individuals entering diapause forsake opportunities for growth as a trade-off for protection against extremely high or low temperatures. In the present version of CLIMEX, only one diapause mechanism can be run at a time. In this model we noted that the cold limits were most important for projecting the potential range of *H*. *armigera*, and the high temperature range limits did not appear to need a diapause mechanism. To account for the cold temperature behaviour in the climate suitability modelling, two versions of the model were run – with- and without a cold diapause mechanism.

#### Irrigation

*Helicoverpa armigera* is found in areas that under natural rainfall conditions extended periods with soil moisture below permanent wilting point, and which appear too arid to support sufficient crop growth and hence population growth for *H*. *armigera*. In these areas it appears likely that *H*. *armigera* is able to survive only in the presence of irrigated crops. To simulate the effects of irrigation the CLIMEX model for *H*. *armigera* was run using 2.5 mm day^−1^ of top-up irrigation throughout the year; a moderate scenario c.f. Yonow & Sutherst [67]. The area over which irrigation is practiced was identified using the Global Irrigated Area V5 (GMIA5) developed by Siebert et al. [68].

### Crop hosts

A composite crop dataset was compiled from the global distribution datasets for a subset of key economic hosts: cotton, sorghum, soybeans, tobacco and wheat in the MapSpaM database [69]. This dataset defines the approximate geographical extent of major crop species, including those that are hosts for *H*. *armigera*.

### Composite risk mapping

The diapause and irrigation scenarios were combined into a fully factorial set of scenarios. Within the irrigation scenarios, the results of the with- and without diapause models were combined, taking the maximum EI value for each cell, reasoning that the allele that was best adapted to the climate within each cell would predominate. The results of this process for each of the irrigation scenarios were combined, drawing on the results for the irrigated scenario where the GMIA5 dataset indicated that irrigation was practiced, and the natural rainfall scenarios elsewhere. For some analyses, results were set to zero where crop hosts were absent according to the MapSpaM dataset.

### Potential cropping impacts in the United States

To assess the potential impacts on cropping in the United States, data on the value of production for the year 2005 for each of the main field crop hosts of *H*. *armigera* growing in the United States (cotton, maize, sorghum, soybeans, tobacco and wheat) were processed from the MapSpaM database. The Global MapSpaM data for Value of Production (VOP) were provided at 5’ gridded spatial resolution. The VOP data for each of the selected hosts were processed as follows:

1. Data were extracted for the conterminous United States,
2. Cell values were aggregated to 10’ resolution to coincide with the CliMond 10’ climate data used for the CLIMEX modelling (CM10 1975H V1.1),
3. Cell values for each of the *H*. *armigera* host crops were aggregated to a single value per 10’ grid cell, and
4. The cell-level host crop production values were intersected with the composite CLIMEX raster (see above).
5. The total value of production potentially exposed to the pest was then derived by summing values for those cells that are climatically suitable to the pest (i.e., where GI_A_ ≥ 10 or EI > 0). The threshold of 10 for GI_A_ was chosen arbitrarily as the minimum climate suitability that could produce noticeable pest impacts.

Our original intention was to use data from the USDA National Agricultural Statistics Service QuickStats dataset (http://www.quickstats.nass.usda.gov). Unfortunately, the available data types from the 2007 agricultural census varied by commodity. For all crops the area harvested (acres) was available. For some crops, the sales values were available, and for others, production was gauged in weight of commodity [either pounds (lbs) or hundredweight (cwt)]. This lack of consistency precluded any meaningful integrated analysis.

Using the MapSpam data, *H*. *armigera* field crop hosts accounted for just under two-thirds of U.S. agricultural output in 2005, some $78.3 billion. Taking account of the potential spatial distribution of *H armigera* reveals that all of this production could be exposed to the pest if its range expanded to the areas indicated as suitable in the CLIMEX model. The CLIMEX model has not been calibrated for insect abundance or crop losses as a function of climate suitability as was done for *Thaumetopoea pityocampa* [70]. Actual losses would likely be substantially lower owing to mitigation efforts and also because it is not likely that all potentially suitable areas would have an infestation in any particular year, or that *H*. *armigera* abundance would cause significant damage.

### United States Border Interceptions

The USDA-APHIS border interception data for “*Helicoverpa*” were extracted for dates between 1 January 1984 and 18 February 2014. The records were filtered for “species = *armigera*.” All of the records were geocoded for the interception port location using the Great Circle Mapper website (http://www.gcmap.com). For a small number of ports for which the International Air Transport Authority (IATA) codes could not be resolved, Google Maps was used to identify geographical coordinates manually based on the location description. These data were mapped by number of independent interceptions at each port, and analysed for trends through time.

## Results

### Potential distribution

The potential distribution, ignoring the distribution of specific crop hosts, based on EI (suitable for persistent populations) and GI_A_ (temporary seasonal range expansion) agrees well with the known distribution of this species (Fig. 1, Figures A and B in S1 File). In Australia the model sensitivity was perfect (1) in relation to the location records for persistent populations. The coarse nature of the distribution data elsewhere precluded calculating model sensitivity.

The most sensitive parameters influencing the modelled range of *H*. *armigera* were the diapause termination temperature (DPT1), followed by the lower soil moisture for growth (SM0). The diapause mechanism is poorly understood, but the soil moisture limits for growth are well characterised by the permanent wilting point for annual vegetation.

### Phenology

**Fig 2:**
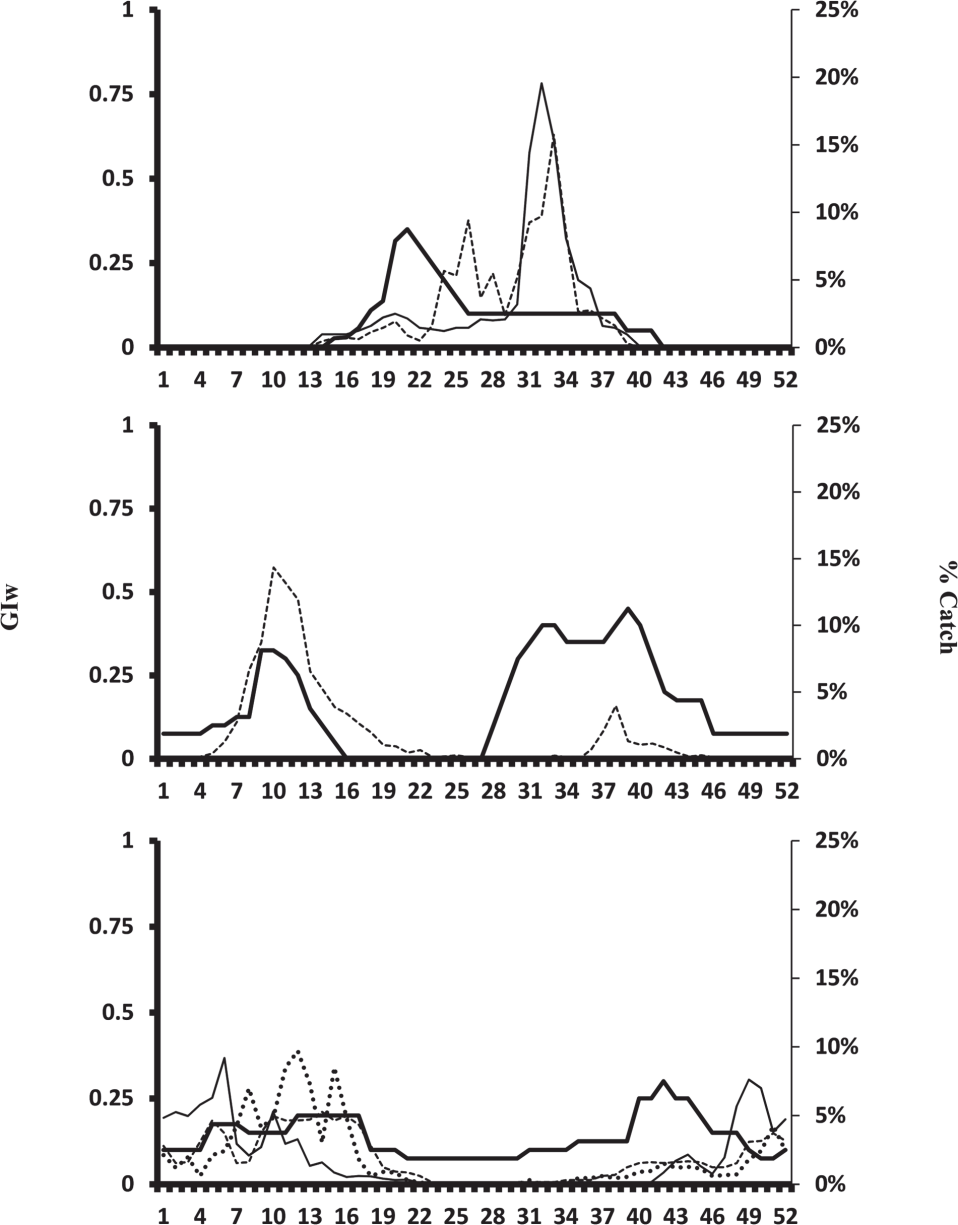
Weekly Growth Index values (Major Y-axis) and various measures of population abundance for *Helicoverpa armigera* (expressed as a % of the total catch, secondary Y-axis) over a year (time in weeks, 1–52) from 1 January. The GI_W_ (thick solid line) is an average of 4 CLIMEX scenarios for *H*. *armigera* (with and without irrigation and with and without diapause) for: (a) Urumqui, Xinjiang province (China) where the two thinner lines are light trap catches in two agricultural landscapes [Aksu county (40° 56’N, 80°27’E) and Maigeiti county (38°53’N, 77°37’E)] [71], (b) Ludhianna (India) where the thinner dashed line is pheromone trap catch of males (V. Dilawari, unpublished data) and (c) Narrabri (Australia) where the solid thin line is for average light trap catches (from [35,46]), and the remaining lines are for male light-trap catch (dashed line) and pheromone trap catch (fine dashed line) (from [72]).

Another weak test of the CLIMEX model is to generate weekly changes in GI_W_ over an average year for a location and compare this “modelled seasonal suitability” with observed seasonal phenology based on say, trapping of adult moths (Fig. 2). We would expect GI_W_ to be a leading indicator of moth population growth rates. For all three sites, trapped moths occur at times which are on average climatically suitable for growth, with variation in seasonal climatic suitability reflected in part in changes in moth numbers (Fig. 2). Moth numbers will of course reflect additional landscape level effects of crop availability, suitability and “management”; the latter usually translates to extensive and intensive spraying of various insecticides [42].

### Distribution and crop hosts

**Fig 3:**
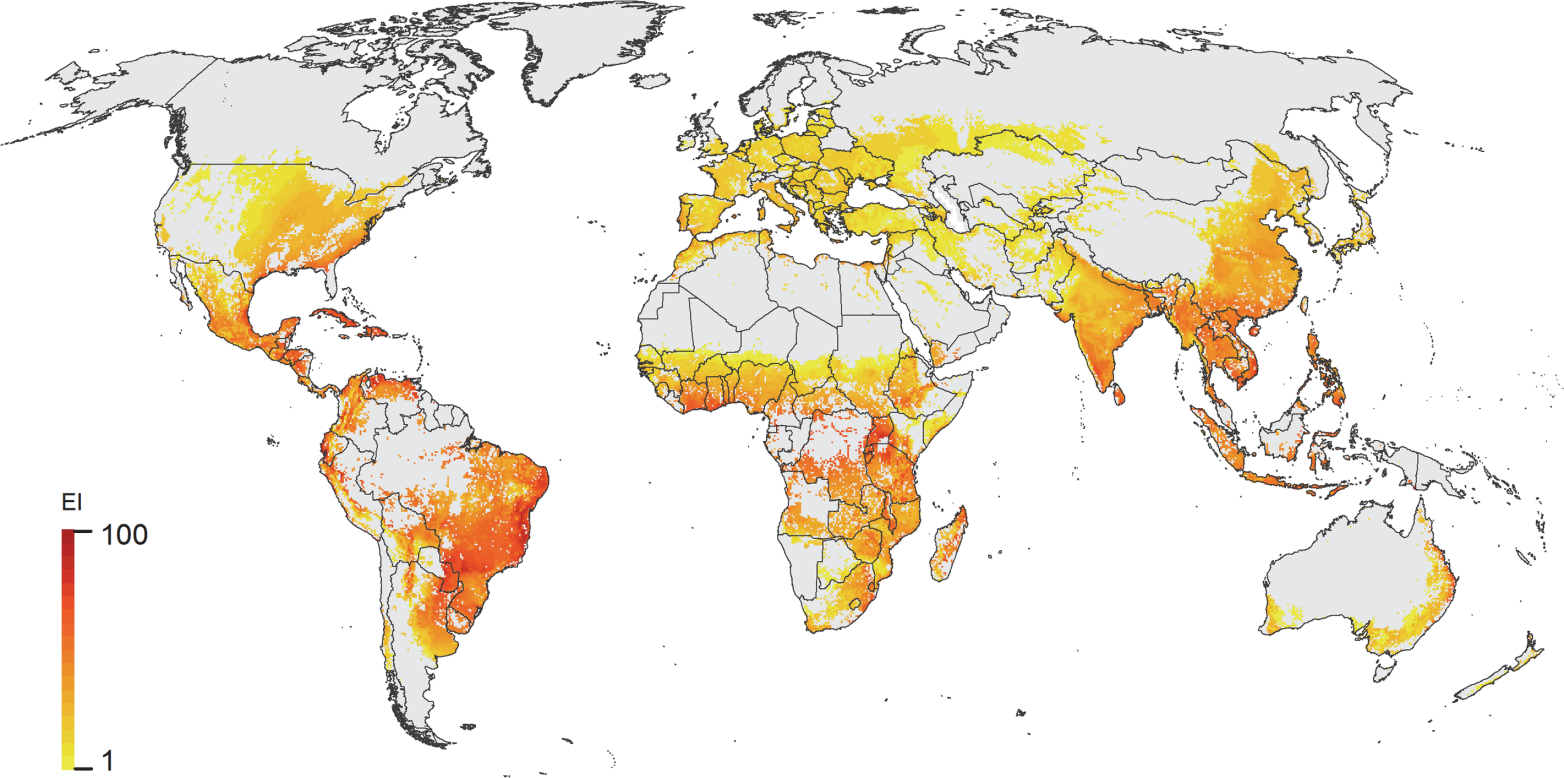
Potential global distribution of *Helicoverpa armigera*, modelled using CLIMEX, taking into account climate suitability, irrigation patterns, and the existence of suitable crop hosts.

When climatic suitability is combined with a cross section of crop hosts for which *H*. *armigera* is a pest (Fig. 3), the potential pest status of the species across its extensive range is apparent. The area of potential establishment of *H*. *armigera* in the New World is extensive (Figs. 1and 3 and Figure B in S1 File).

**Fig 4:**
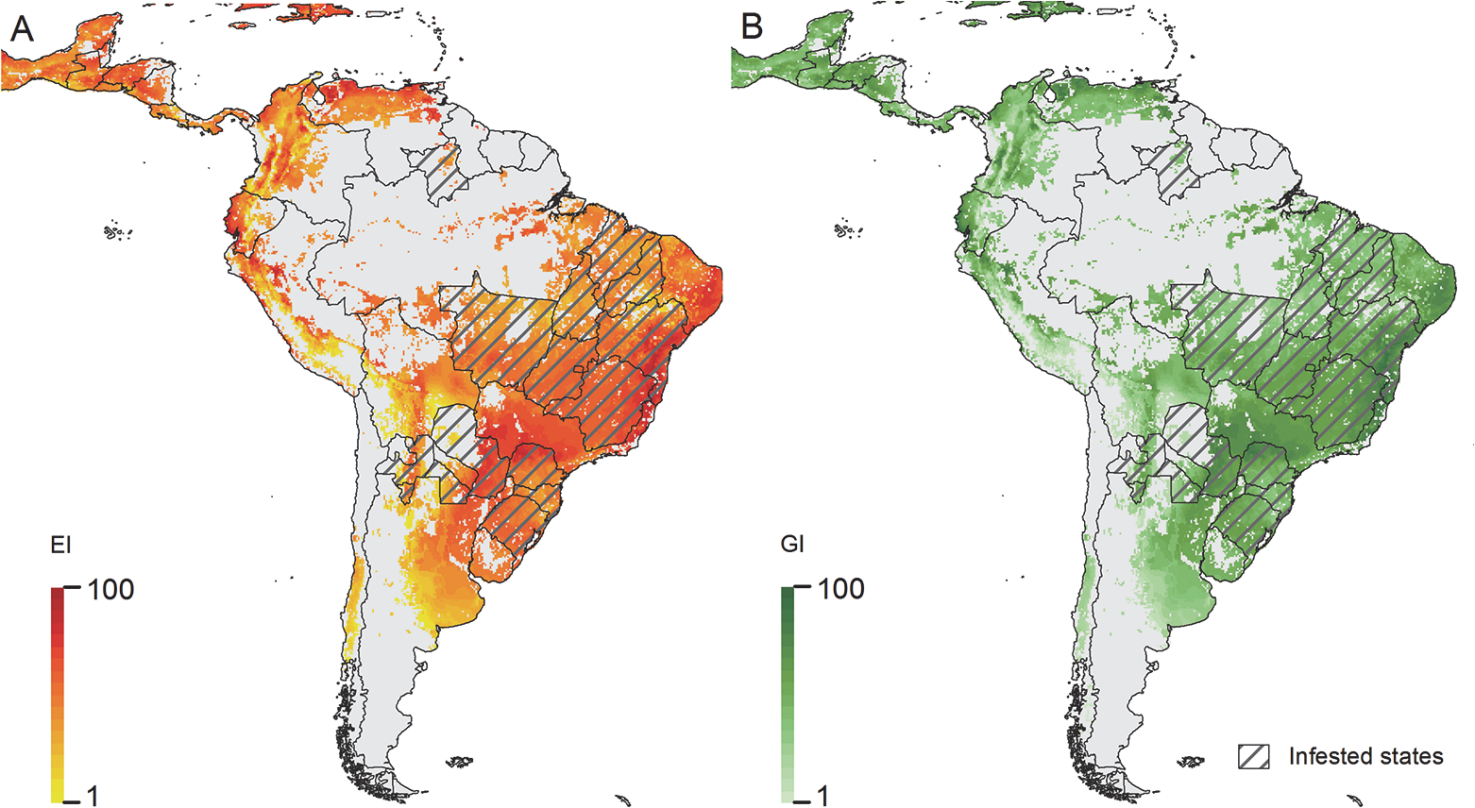
Climate suitability for *Helicoverpa armigera* in South America, modelled using CLIMEX, taking into account irrigation patterns and the existence of suitable crop hosts. A) Ecoclimatic Index (EI), indicating favourability for persistence, B) Annual Growth Index (GI_A_), indicating the potential for population growth.

*Helicoverpa armigera* has established in Brazil in an area with a generally high EI (Fig. 4). Populations have been high there, impacting significantly on key crops such as corn, soybeans, tomatoes and cotton (to name a few) [5]. The species has already been detected in Argentina, Bolivia, Paraguay, Uraguay and Puerto Rico [7,8,9], and given the extensive production of suitable host crops, year round in many cases with irrigation, the high levels of abundance and the propensity of the moth to move on wind systems, we suspect the species will continue to spread further in South and Central America (Figs. 1 and 4).

### United States Border Interceptions

**Fig 5:**
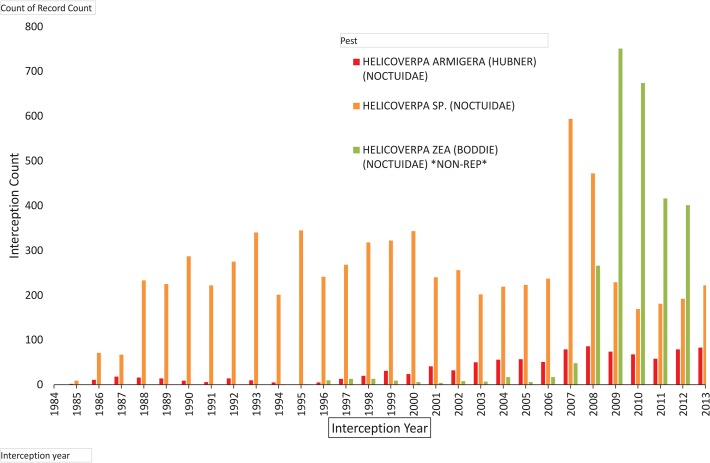
Border interception frequency for selected Heliothine moths in the United States (1984–2013) (source USDA-APHIS). Other species not shown due to low frequency of interceptions: *Helicoverpa assulta*, *H*. *gelotopoeon* and *H*. *punctigera*.

Since 1984, *H*. *armigera* has been positively identified in 1 017 border interceptions in mainland United States. The difficulty in distinguishing *H*. *armigera* from its congeners (especially from *H*. *zea*) using morphological characteristics, and the large number of interceptions (7 203) keyed to *Helicoverpa* means that the real number of *H*. *armigera* intercepts is likely to be even greater (Table 2). In the period being considered, the overall interceptions remained fairly steady until 2007, when *H*. *zea* started being trapped in remarkably large numbers, and the interception rate of *H*. *armigera* also started to increase steadily (Fig. 5).

**Table 2:** Total port interceptions for *Helicoverpa* species in the United States from 1984 to 2013.

Year	*Helicoverpa armigera* (HUBNER)	*Helicoverpa assulta* (GUENEE)	*Helicoverpa gelotopoeon* (DYAR)	*Helicoverpa punctigera* (WALLENGREN)	*Helicoverpa* Sp.	Helicoverpa zea (BODDIE)	Grand Total
1984	5						5
1985	2				9		11
1986	11				71		82
1987	18				67		85
1988	16				233		249
1989	14				225		239
1990	9				287		296
1991	6				222		228
1992	14				275		289
1993	10				340		350
1994	5				201	1	207
1995					345	1	346
1996	5				241	10	256
1997	13				268	13	294
1998	20	1			318	13	352
1999	31			2	322	9	364
2000	24	11			343	6	384
2001	41	4			240	4	289
2002	32	3			256	8	299
2003	50	1	1		202	7	261
2004	56				219	17	292
2005	57	1	2		223	6	289
2006	51	1			237	17	306
2007	79	2	1		594	48	724
2008	86				472	266	824
2009	74				229	751	1054
2010	68				169	674	911
2011	58				181	416	655
2012	79				192	401	672
2013	83				222	335	640
**Grand Total**	**1 017**	**24**	**4**	**2**	**7 203**	**3 003**	**11 253**

Source, USDA APHIS inspection database.

### Risk of introduction, establishment and potential impact in North America

**Fig 6:**
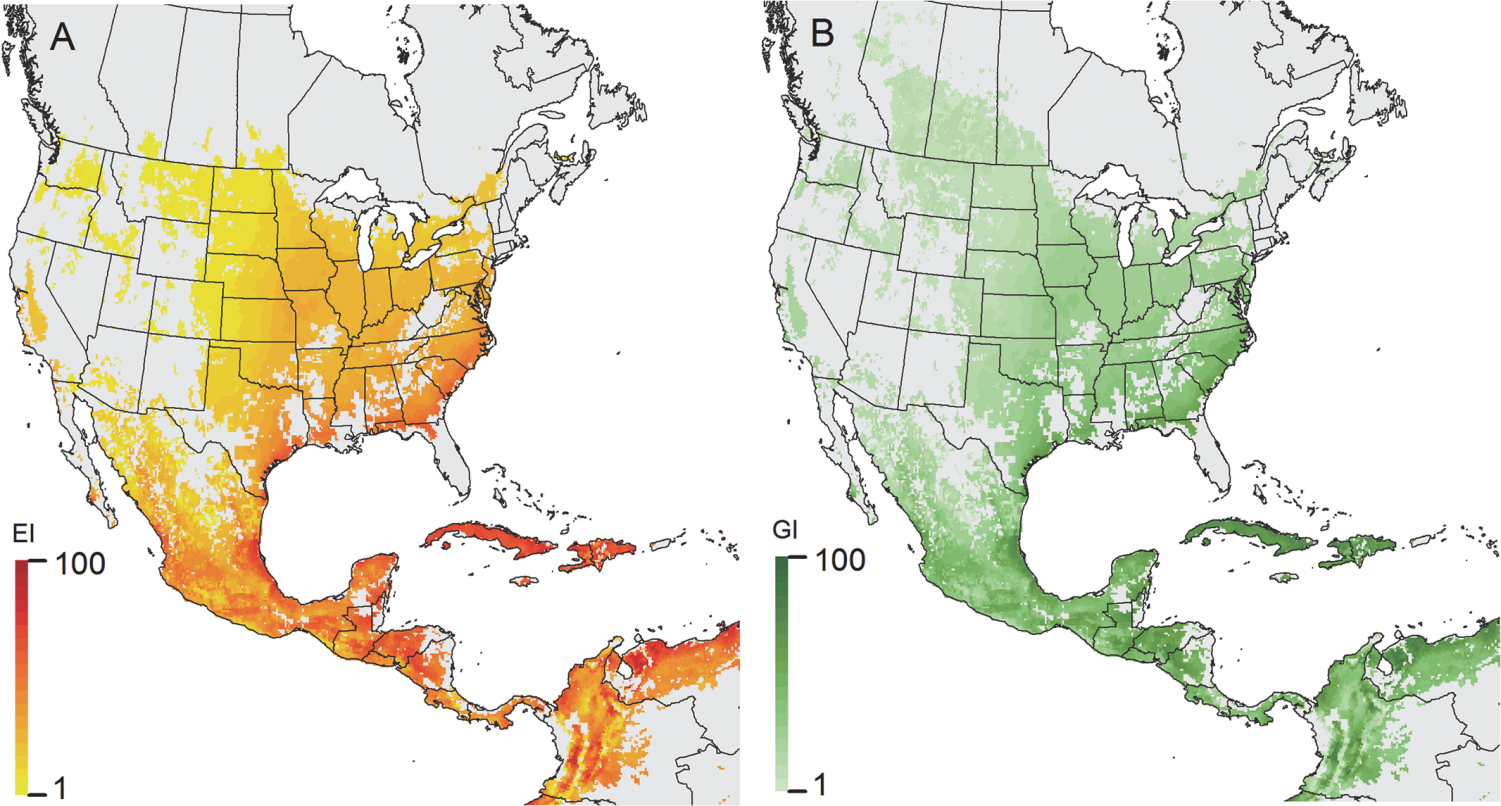
Climate suitability for *Helicoverpa armigera* in North America, modelled using CLIMEX, taking into account irrigation patterns and the existence of suitable crop hosts in the MapSpaM dataset. A) Ecoclimatic Index (EI), indicating favourability for persistence, B) Annual Growth Index (GI_A_), indicating the potential for population growth.

**Fig 7:**
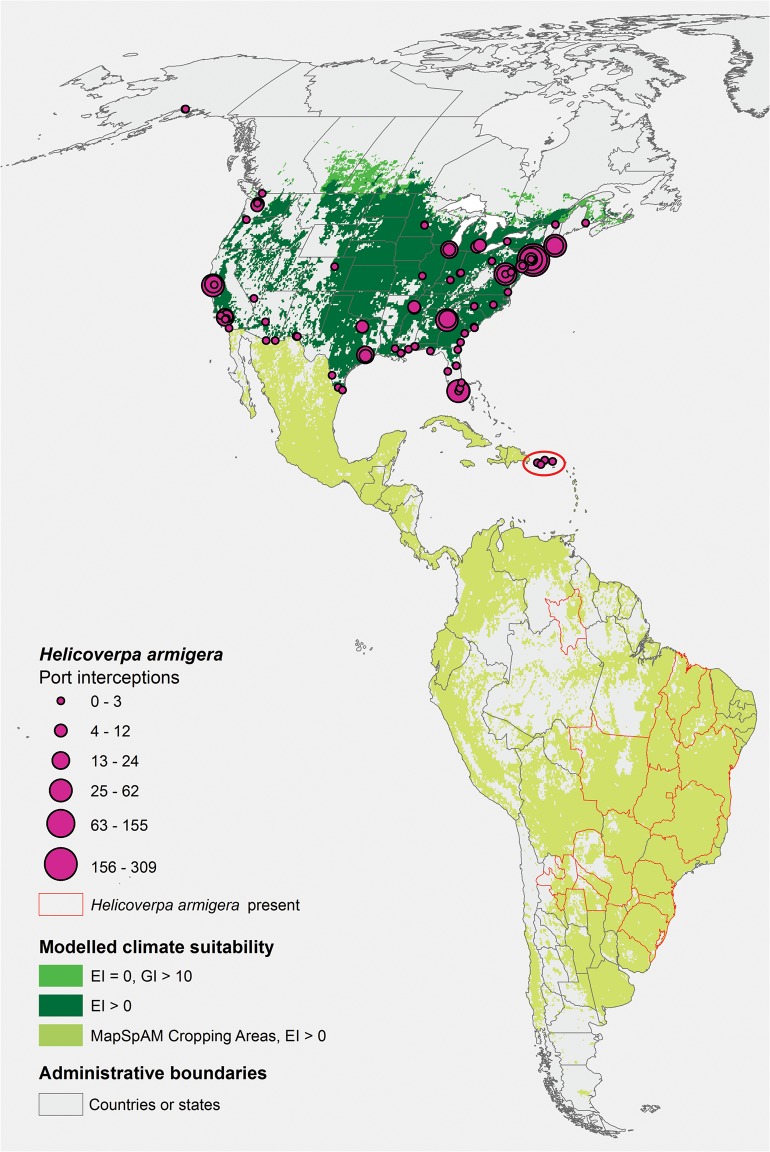
The invasion threats to North America by *Helicoverpa armigera*. South American locations known to be infested by *H*. *armigera* are indicated by red polygon outlines. Cropping areas in Central and South America suitable for establishment (EI positive under appropriate irrigation and natural rainfall scenarios) are indicated in pale green. In North America, areas suitable for establishment are indicated in dark green. Areas suitable for seasonal migration-based impacts are indicated in bright green.

Suitable climate and extensive areas of host crop plants are readily available in North America (Fig. 6). Whilst there have been a large number of border interceptions of *H*. *armigera* within the cropping zones, so far, it appears that there have been no establishment events or post-border detections in the United States. There is contiguous suitable habitat across the Isthmus of Panama, and the Caribbean islands are also mostly climatically suitable for *H*. *armigera* (Fig. 7). Should it spread further into these areas it is likely that *H*. *armigera* would be capable of invading the United States and Canada through natural dispersal on a regular basis.

Using a reasonable threshold of 10 for EI and GI_A_ reveals that the majority of the crop values for the major hosts are at risk (US$71,755 million from established populations, and US$112 million from transient populations, Table 3). Even considering just the most climatically optimal locations suggests that US$843 million worth of crops is under a substantial threat. Values for individual crops and for various scenarios (Table 3), indicate that 65% of total U.S. agricultural output is potentially exposed to *H*. *armigera*. Clearly, this pest warrants attention.

**Table 3:** Value of agricultural production in 2005 for the conterminous United States at risk from invasion by *Helicoverpa armigera* at different levels of climate suitability modelled using CLIMEX.

Value of Production (Million US$, 2005)						
Crop	Total U.S. Value of Crop	A	B	C	D	E	F
		Establishment + seasonal presence	Establishment	Establishment and pest impacts	Optimal climate	Seasonal presence	Seasonal population growth
		EI > 0 and GI_A_ > 0	EI > 0	EI > 10	EI > 50	EI = 0 AND GI_A_ > 0	EI = 0 AND GI_A_ > 10
Cotton	4,078	4,078	4,078	3,968	292	-	-
Maize	40,121	40,121	40,105	38,208	207	16	14
Sorghum	1,463	1,463	1,463	1,305	115	8	-
Soybeans	23,362	23,362	23,356	22,922	131	7	5
Tobacco	543	543	543	543	66	-	-
Wheat	8686	8,686	8,477	4,808	31	205	93
Total	78,254	78,254	78,022	71,755	843	228	112

Production values have been taken from MapSpaM data [69].

EI is the Ecoclimatic Index, GI_A_ is the Annual Growth Index.

## Discussion

### Global potential distribution

With its discovery in Brazil, *H*. *armigera* is now present in all continents that are climatically suitable, except for North America. Given the pest threat it poses to such a wide variety of economically important crops, it is perhaps surprising that there is so little geographically precise data on its distribution, abundance, and crop yield impacts. Similarly, its phenology is relatively poorly understood, particularly with respect to the factors controlling the pattern of diapause. Given its notable migratory ability and short generation time, the range margins of *H*. *armigera* are indistinct particularly with respect to the regions in which it can overwinter. These uncertainties combine to limit our ability to model the potential distribution of *H*. *armigera* with great precision. Nonetheless, the modelled potential distribution (Fig. 3) accords well with the known distribution (Fig. 1). Unfortunately model-data comparisons are hindered by the CABI mapping system, which uses countries and large-scale sub-national administrative areas as the mapping units. For example, CABI claims that *H*. *armigera* is widespread throughout North African countries covering the Sahara Desert, when it seems obvious that its presence would be restricted to the narrow band of peri-coastal land which experiences a Mediterranean climate and is capable of supporting cropping activities, rather than the more xeric interior, except perhaps following localised seasonal rainfall.

The presence of *H*. *armigera* in northern Australia outside the cropping zone (Figs. 1 and 3 and Figure A in S1 File) highlights the fact that its hosts and climatic potential range extend well outside of the current cropping zone. *Helicoverpa armigera* is climatically well suited to the agricultural regions of South America, and unsurprisingly, it is spreading rapidly throughout Argentina, Bolivia, Brazil, Paraguay and Uruguay, and most recently into Cost Rica and Puerto Rico (Fig. 1). Further spread westward and northwards through Peru, Ecuador, and Columbia, and northwards through Venezuela seems almost inevitable. The successful establishment and spread of the species seems to have been unhindered by the presence of closely related heliothines, ostensibly in the same or similar niche. Continued spread through South America may well be reminiscent of the spread of Africanised bees, which dispersed rapidly throughout the continent crossing into Central America and onto the Southern United States in less than 50 years [73]. Unlike *H*. *armigera*, the bees were not particularly migratory.

Cropping regions throughout the United States appear climatically suited to a greater or lesser extent, being capable of supporting established populations of *H*. *armigera*, particularly in the south (Fig. 6A). Given the known migratory ability of *H*. *armigera* [15,74], and its similar ecology to *H*. *zea*, it is likely that if established in the southern USA, *H*. *armigera* moths would be predisposed to annual long-distance migration northward, via the classic North American Low-level jet (LLJ) phenomenon (east of the Rocky Mountains), that is common during summer months [75,76,77]. Consequently, *H*. *armigera* populations carrying pesticide resistance genes would also facilitate exploitation of Midwestern and northern USA crops, as observed with *H*. *zea* [78]. The model presented here suggests however that the exploitation of the northern cropping regions in the United States may not be as dependent on the annual migration of moths from more clement cropping regions (Fig. 6A). Conversely, under current climatic conditions the majority of Canada’s cropping regions (Saskatchewan and Alberta) appear suitable for supporting only transient seasonal populations (Fig. 6A-B). The importance of other transient migratory pests in this region, such as Diamondback moth (*Plutella xylostella*) [79] serves to underscore the potential pest impacts of transient pests originating in the southern United States and Mexico into these valuable high-latitude cropping regions.

The potential range limits for persistent populations of *H*. *armigera* in cold regions are not well defined geographically. Recent research suggests that the pupal cold tolerance limits for *H*. *zea* under current climate conditions in the United States may be limited to the 40^th^ parallel in the interior of the continent [63]. Such limits may, or may not apply to *H*. *armigera;* it is possible that since the genetic separation, *H*. *armigera* may have evolved additional cold tolerance. It is also plausible that *H*. *armigera* and *H*. *zea* share the same or similar cold tolerance mechanisms and limits. Either way, in terms of the risk assessment, there may be little that distinguishes between areas capable of supporting overwintering pupae, and those that experience seasonal immigrant flights of *H*. *armigera* during the cropping season.

### Economic impacts

The total value of crop production in the USA at risk from *H*. *armigera*, nearly $72 billion *per annum*, is attention grabbing. There is presently no means of calibrating a function relating climate suitability for *H*. *armigera* to crop damage, such as has been done for *Puccina graminis* in wheat [80], *Thaumetopoea pityocampa* in *Pinus* spp., [70], or even the semi-quantitative methods of Pinkard *et al*. for estimating the management significance of *Mycosphaerella* leaf disease in *Eucalyptus* spp. plantations [81]. Thus, it is presently impossible to estimate what fraction of the total value of production of the suitable crops in the USA might be at threat from *H*. *armigera* should it invade the USA. Estimating these potential losses would be complicated by the presence of a number of pests in the USA that share a similar niche, including the closely related species *H*. *zea*. The existence of these pests means that at least some of the control costs for *H*. *armigera* would already be included in the present pest control costs expended by agricultural producers.

It may be worthwhile considering qualitatively the management of *H*. *armigera* overseas, as a means of forewarning production systems in North America. Management of *H*. *armigera* has relied on insecticides, and it has repeatedly developed resistance to all chemical classes used to date [42,82], with some major disasters in both tropical [83] and temperate regions [84]. It was in part to combat *H*. *armigera* that cotton varieties were genetically engineered to express various Bt Cry toxin genes and deployed across the Old World. To date, with Insect Resistance Management (IRM) strategies, GM cotton has been very successful in Australia [85] although resistance is on the rise [86]. In North and South America, GM crops expressing Cry toxins include corn and soybean in addition to cotton. They were introduced to control native lepidopteran pests such as *H*. *zea*, *Heliothis virescens* and *Spodoptera frugiperda*, and the Australian experience suggests that they may well prove effective against *H*. *armigera* when combined with a well-designed management strategy. Globally, over four hundred million hectares of Bt crops have been planted, and resistance in a number of species has been on the increase [87].

In Australia, the widespread replacement of conventional cotton with plants genetically modified to express various *Bacillus thuringiensis* (Bt) toxins has effectively turned one major crop into a population “sink” for *H*. *armigera* across whole landscapes where cotton is grown [39]. Currently Bt cotton comprises nearly 90% of all cotton crops in Australia [42], as it does in other parts of the world [17]. This landscape-level change began in the late 1990’s, with the introduction of one-gene cotton transformations (INGARD), followed by two-gene Bollgard II in 2004. An IRM Strategy that relies on refuges to produce susceptible moths [71] has delayed the development of resistance to the technology [88]. Nevertheless variation in population size due in part to climate [12] can put pressure on pest management systems [42]. Overall, the overseas experiences suggest therefore that *H*. *armigera* is not an “Armageddon pest”, but rather a problem that requires integrated management systems that are themselves managed to slow the development of pesticide resistance, and to respond to changing pesticide resistance patterns.

### Niche model interpretation and limitations (including comparison to previous model)

Our CLIMEX model agrees with the available distribution data, development rate experiments, and phenological observations. However, the existing distribution data is mostly imprecise, and hence the known and potential distribution maps should be treated as indicative. The diapause and cold stress functions were fitted to the best of our ability using the available data, but we are not confident of their precision, suggesting these are possibly priority areas for additional research. Our confidence in the modelled potential distribution presented here is bolstered by the similar distribution of the closely-related *H*. *zea* in the United States, which likely shares a similar niche [5].

The previous CLIMEX model of Zalucki & Furlong [12] indicated a positive EI value for large areas of arid and semi-arid habitat in Central Australia, where the climate is only suitable for *H*. *armigera* during favourable seasons and years. The poleward distribution limits of the present model match the known distribution limits better than the Zalucki & Furlong model. Compared with the coarse biome-comparison methods of Venette *et al*. [10], the present model indicates a more extensive climatically suitable area for *H*. *armigera*, with suitable habitat extending into colder continental climates, and more xeric regions than the biome analysis. The northern limits in temperate regions of the USA (eastern third) are similar, though it is not clear whether the limits in the Venette *et al*. report [10] are due to the USA border or the biome border.

Whilst the modification of the Zalucki and Furlong model may be interpreted as an implied criticism of the original model, the changes we implement here are more of a reframing or redefinition of the model. Whereas the original modelling attempted to indicate both persistent and ephemeral habitat through a positive EI value, in the present model we define these two types of habitat explicitly using a combination of EI and GI_A_ values.

The various data elements used in this analysis span a range of temporal frames. The climate is centred on 1975, the crop distribution data on 2000 and the value of production and irrigation data on 2005. These temporal mismatches should have minimal impact on the analytical results. The CLIMEX model parameters were fitted to recently updated distribution data. To the extent that the climate has changed since the 1975 average conditions, the parameters have been automatically adjusted to compensate.

### Biosecurity implications for North America

With its establishment in South and Central America, several invasion routes into North America now appear open to *H*. *armigera*: 1) direct air or sea transport, 2) natural spread across the Isthmus of Panama into the high-elevation cropping areas of Mexico and then A) natural migration in the spring or B) land transport into the United States, and 3) further island-hopping across the Caribbean islands. In recent times *H*. *armigera* has been detected frequently associated with goods transported into the United States (Table 2, Fig. 6), yet so far apparently failed to establish resident populations outside the quarantine border facilities. It seems unlikely that the border interceptions included every introduced specimen of *H*. *armigera*. Whilst there is no direct evidence of the detection rate in this case, based on the few studies available (e.g., [89]), we presume that despite the best efforts of the US border biosecurity agencies, a significant number of *H*. *armigera* are transported directly into the United States associated with fresh produce. The failure so far of *H*. *armigera* to establish there is likely therefore due to inhospitable conditions post-arrival through the consumer product delivery process, or asynchrony of life-stages with the climatic seasons upon arrival. The vast majority of border detections of *H*. *armigera* in the United States have been associated with cut flowers, mint and basil (Table B in S1 File). The post-arrival treatment and final uses of these commodities may limit the potential for *H*. *armigera* to establish in the United States. Nonetheless, the alarming frequency of *H*. *armigera* intercepts in the United States points to significant problems with the sanitary precautions implemented prior to the export of these commodities. If it has not happened already, an increase in pheromone trapping surveillance around ports of entry and southern US cropping districts might appear warranted to enable biosecurity responses to be implemented as soon as possible. However, this might be a futile effort if *H*. *armigera* can establish in locations in Central America or the Caribbean from where it can disperse into the USA naturally.

Another reason for the failure of *H*. *armigera* to establish in the United States in recent times is perhaps due to founder effects; the small numbers of individuals in each introduced population [90]. In the relatively short-term, the presence and continued spread of *H*. *armigera* in South and Central America may facilitate its invasion along the Central American isthmus and the Caribbean islands, from which further natural dispersal into North America is clearly possible.

Whilst there are several closely-related heliothines already established in the United States, none have the broadscale pattern of pesticide resistance observed in *H*. *armigera*. The threat posed to a broad range of economically important crops suggests that some significant preparedness activities may be warranted. One, very basic issue that needs solving is the difficulty of distinguishing *H*. *armigera* from the native *H*. *zea* (North and South America) from which it is estimated to have diverged from *H*. *armigera* only approximately 1.5–2 million years ago [91] and *H*. *gelotopoeon* (South America). Traditional taxonomic methods require very specialised skills and molecular tools are likely to be required. Behere *et al*. [92] have reported using two partial mitochondrial DNA genes (COI, Cytb) as markers to differentiate between *H*. *armigera*, *H*. *punctigera*, *H*. *assulta* and *H*. *zea*. Improving this method to include other *Helicoverpa* species (e.g., *H*. *gelotopoeon*, *H*. *minuta*) may also be desirable. A further complication is the fact that *H*. *armigera* and *H*. *zea* have been shown to hybridise in the laboratory and could well be hybridising in the field [14,93]. It is unclear if *H*. *armigera* can hybridise with other endemic heliothine species, such as with *H*. *gelotopoeon*. The mtDNA marker method of species differentiation of Behere *et al*. will not be able to correctly identify *zea-armigera* hybrids that resulted from mating between a female *H*. *zea* and a male *H*. *armigera*; these hybrids would be incorrectly identified as *H*. *zea* [92]. The prospect of hybrids, indistinguishable using morphological and standard molecular tools from *H*. *zea*, but containing *H*. *armigera* resistance genes is one that would dramatically complicate control and/or eradication initiatives.

Slowing the spread of *H*. *armigera* throughout South America, Central America and the Caribbean would seem to be a prudent goal, though given the previous pesticide resistance patterns, may be problematical. Given the long lead times, initiating classical and inundative biological control programmes, sterile insect techniques, large-scale pheromone trapping, trap crops at borders would seem prudent activities to initiate promptly. It may even be in the interests of the North American countries to co-invest in biological control and spread monitoring programmes in Central America and the Caribbean. Reduced populations of *H*. *armigera* in these regions could translate into reduced rates of spread into North America. In Brazil, researchers have been dedicated to identifying potential biological control agents for *H*. *armigera* and the adoption of inundative biological control using *Trichogramma* spp., viruses and Bt bioinsecticides have been increased since the detection of *H*. *armigera* in the 2012–2013 cropping season [94].

The incursion of *H*. *armigera* into South America and its subsequent spread into Central America and the Caribbean has changed the nature of the invasion risk to North America fundamentally. Previously, the perceived threat was from goods and services being transported into North America, from various locations worldwide, including South and Central America and the Caribbean. Now there are two likely natural dispersal pathways, via the land bridge between North and South America, or via island-hopping across the Caribbean. Not only has the dispersal route changed, but so has the nature of the invasion pathway. These changes have important ramifications for the constraints on biosecurity response actions. If an incursion happened as a result of an isolated, low frequency human-mediated dispersal event, then an eradication could feasibly be contemplated. However, a central tenet in the definition of an eradication of an unwanted organism is that there is low probability of re-invasion. If the USA is invaded by *H*. *armigera* from either Central America or the Caribbean, then, as demonstrated by many migratory insects such as *P*. *xylostella* and *Danaus plexippus*, it is highly likely that it will be capable of re-invading on a regular basis. Another potential problem lies in the close association between *H*. *zea* (usually considered a native North American pest) and *H*. *armigera*, which would make eradication of one without the other practically impossible. These complications suggest that there may be limited available responses such as:

1. trying to slow the spread of *H*. *armigera* through Central America and the Caribbean,
2. preparing agricultural producers in the USA with information on the spread of *H*. *armigera*, and explaining why an eradication effort would be imprudent and impractical,
3. monitoring the pesticide resistance profiles of *H*. *armigera* populations as it spreads through Central America and the Caribbean, with a view to adjusting the integrated pest management recommendations in the southern USA.

During the preparation of this paper, the distribution maps for *H*. *armigera* had to be revised several times to accommodate fresh reports of its spread. There are no apparent barriers to its spread through Central America to the point where it could migrate into the agricultural regions of North America. Its rapid spread into the Caribbean also reflects its strong migratory abilities. The spread of *H*. *armigera* into North America, not only looks to be a matter of time, but a short one at that!

## Supporting Information

S1 File This file contains the supporting information for this article including Figures A and B and Tables A and B. Figure A, Climate suitability for *Helicoverpa armigera* in Australia modelled using CLIMEX for A) Establishment, and B) Population growth in relation to its known distribution. Point locations indicate the goodness of fit of the model. Figure B, Global climate suitability for *Helicoverpa armigera* modelled using CLIMEX for A) Establishment, and B) Population growth in relation to its known distribution. Point locations and shaded areas indicate the goodness of fit of the model. Table A, CLIMEX Parameter Sensitivity for *Helicoverpa armigera*. Table B, Frequency of interceptions of *Helicoverpa armigera* in the United States by inspected host.
(DOCX)
