# Correction: The Potential Distribution of Invading *Helicoverpa armigera* in North America: Is It Just a Matter of Time?

**DOI:** 10.1371/journal.pone.0133224

**Published:** 2015-07-16

**Authors:** Darren J. Kriticos, Noboru Ota, William D. Hutchison, Jason Beddow, Tom Walsh, Wee Tek Tay, Daniel M. Borchert, Silvana V. Paula-Moraes, Cecília Czepak, Myron P. Zalucki

The eighth author’s name is spelled incorrectly. The correct name is: Silvana V. Paula-Moraes.

One of the affiliations for the third and fourth authors is not indicated. William D. Hutchison and Jason Beddow are both additionally affiliated with: Minnesota's Discovery, Research and Innovation Economy (MNDrive) Initiative, University of Minnesota, St. Paul, Minnesota, United States of America.
