# Supplementary material for: The Potential Distribution of Invading Helicoverpa armigera in North America: Is It Just a Matter of Time?
Source: PLoS One. 2015 Mar 18;10(3):e0119618. doi: 10.1371/journal.pone.0119618 (PMC4364701; doi:10.1371/journal.pone.0119618)
Supplement: S1 File — Figure A, Climate suitability for Helicoverpa armigera in Australia modelled using CLIMEX for A) Establishment, and B) Population growth in relation to its known distribution. Point locations indicate the goodness of fit of the model. Figure B, Global climate suitability for Helicoverpa armigera modelled using CLIMEX for A) Establishment, and B) Population growth in relation to its known distribution. Point locations and shaded areas indicate the goodness of fit of the model. Table A, CLIMEX Parameter Sensitivity for Helicoverpa armigera. Table B, Frequency of interceptions of Helicoverpa armigera in the United States by inspected host. (DOCX) [file pone.0119618.s001.docx]

# The potential distribution of invading *Helicoverpa armigera* in the Americas: Is it just a matter of time?

**Supplementary Material**

Darren J. Kriticos, Noboru Ota, William D. Hutchison, Jason Beddow, Tom Walsh, Wee Tek Tay, Daniel Borchert, Silvana V. Paula-Moreas, Cecília Czepak and Myron P. Zalucki

**List of Tables and Figures in the Supplementary Online Material**

**Figure A** Climate suitability for *Helicoverpa armigera* in Australia modelled using CLIMEX for A) Establishment, and B) Population growth in relation to its known distribution. Point locations indicate the goodness of fit of the model.

**Figure B** Global climate suitability for *Helicoverpa armigera* modelled using CLIMEX for A) Establishment, and B) Population growth in relation to its known distribution. Point locations and shaded areas indicate the goodness of fit of the model.

**Table A** CLIMEX Parameter Sensitivity for *Helicoverpa armigera*.

**Table B.** Frequency of interceptions of *Helicoverpa armigera* in the United States by inspected host.


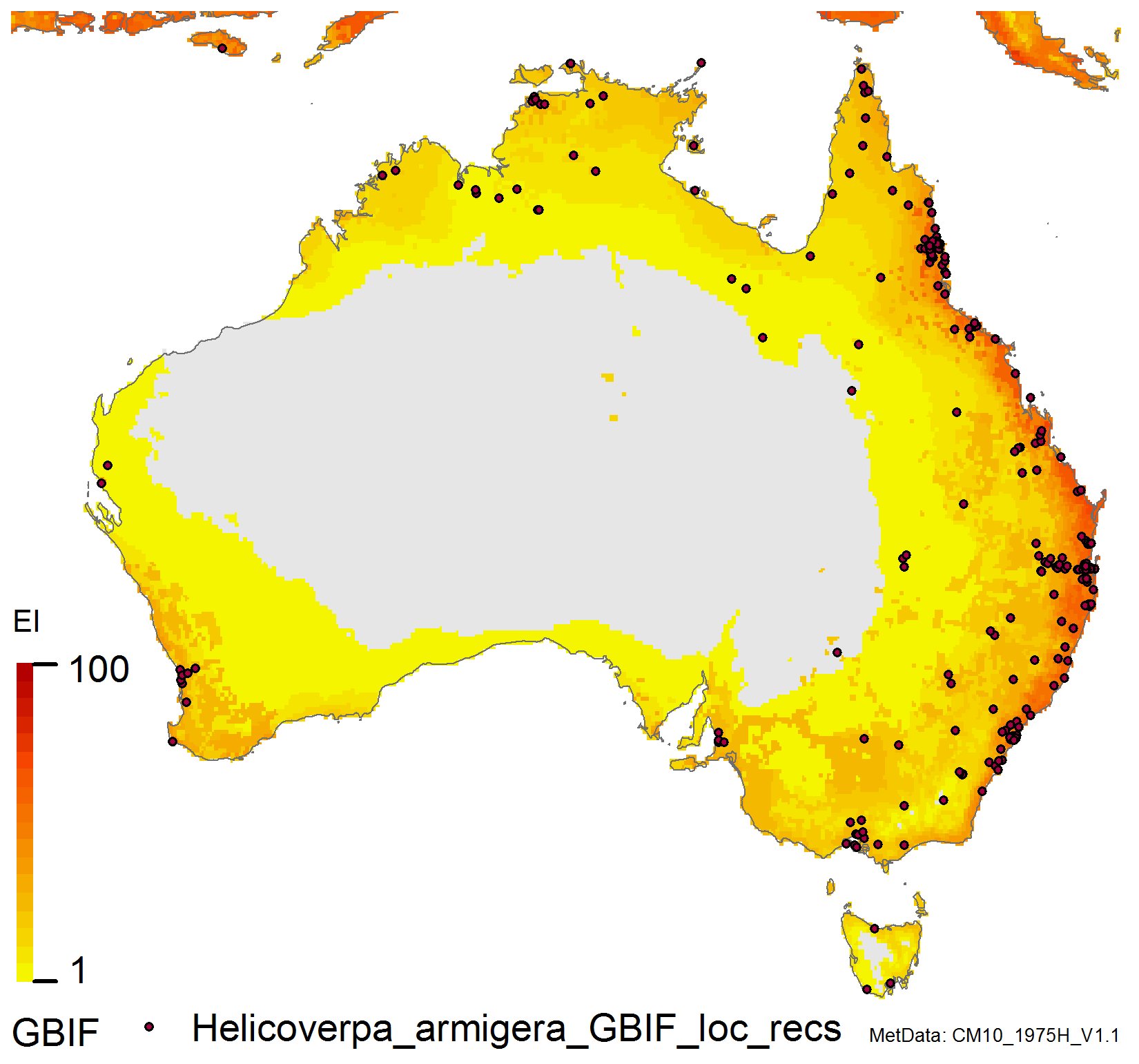


A


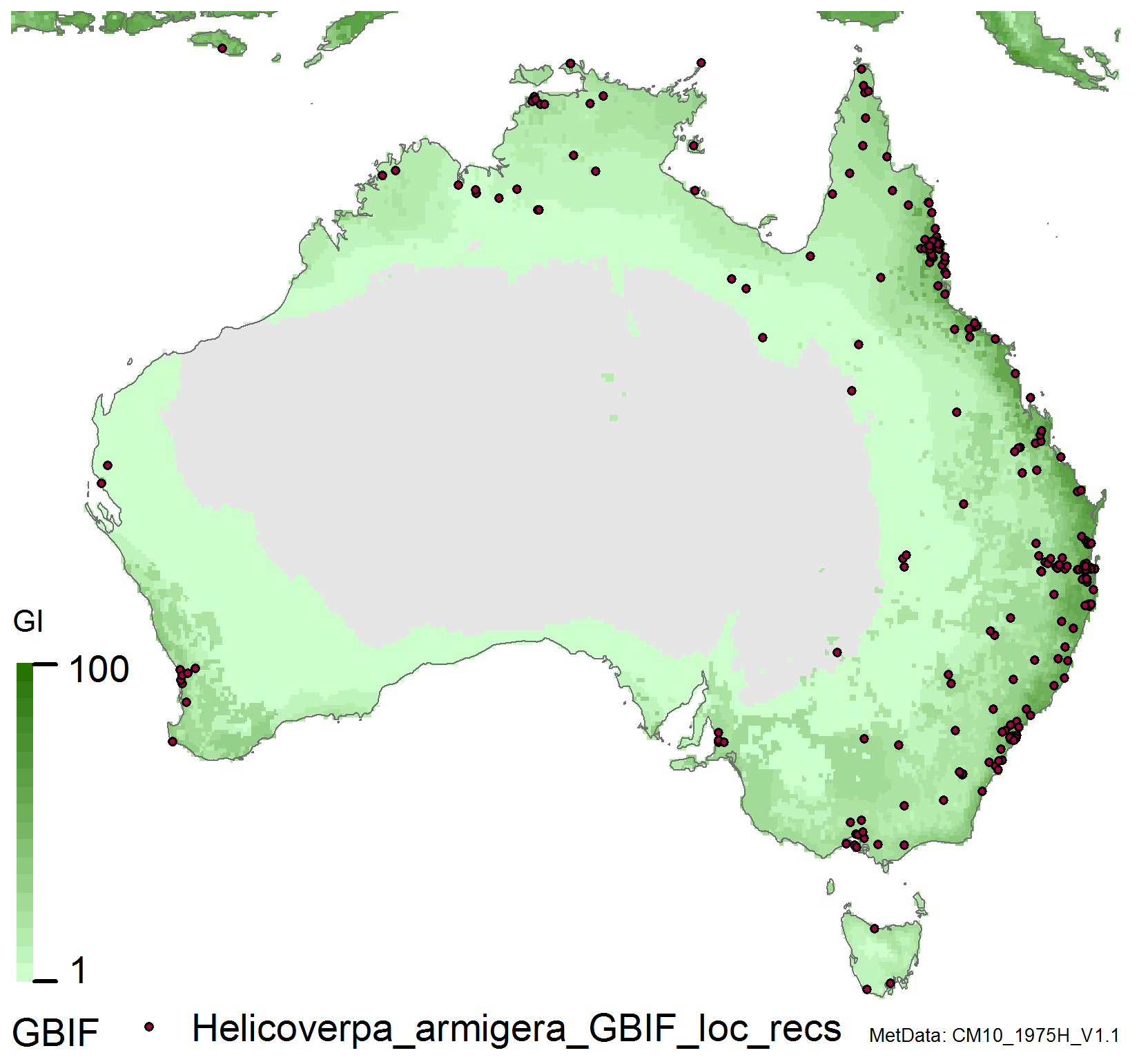


B

**Figure A.** Climate suitability for *Helicoverpa armigera* in Australia modelled using CLIMEX for A) Establishment, and B) Population growth in relation to its known distribution. Point locations indicate the goodness of fit of the model.


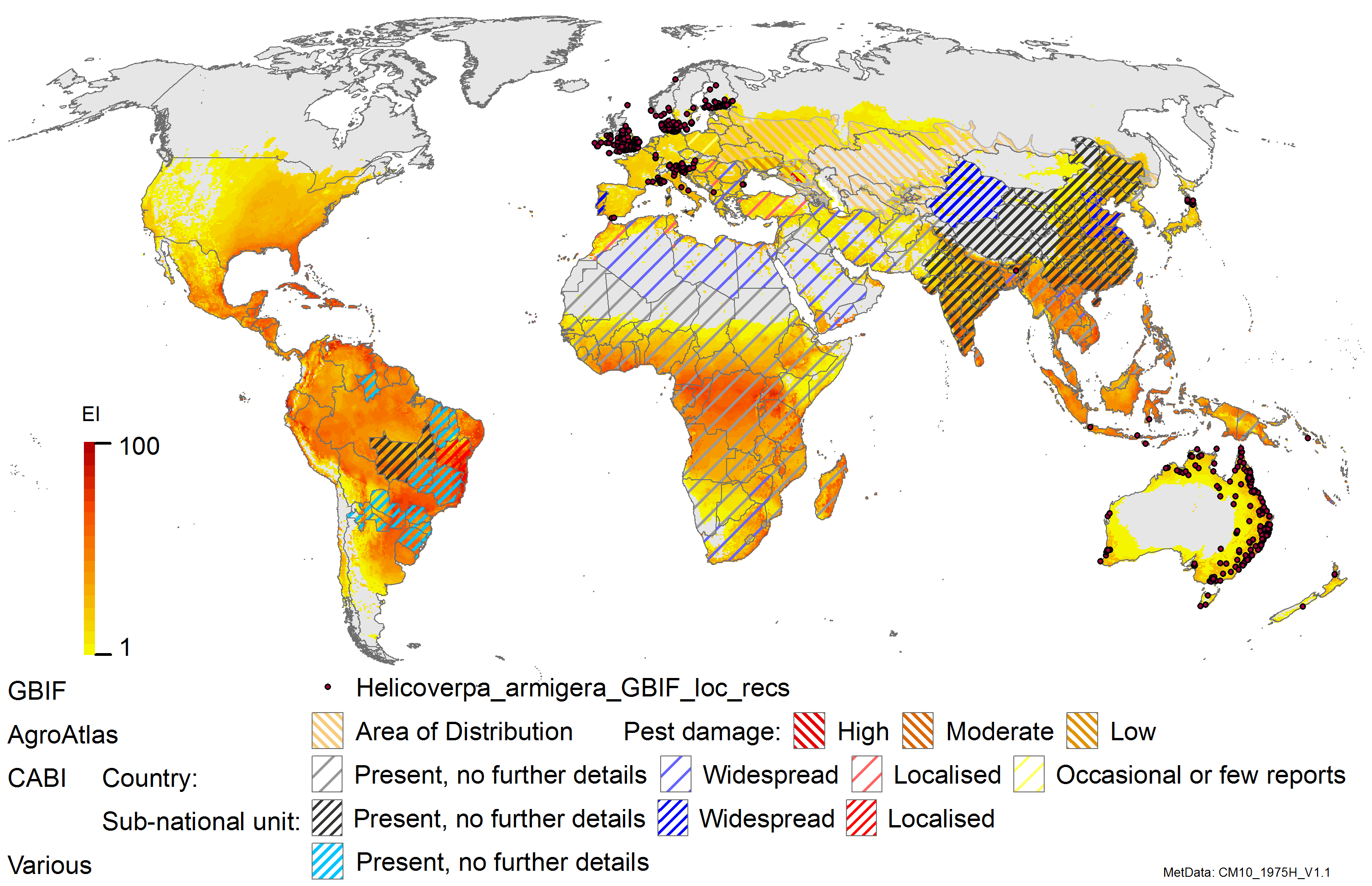

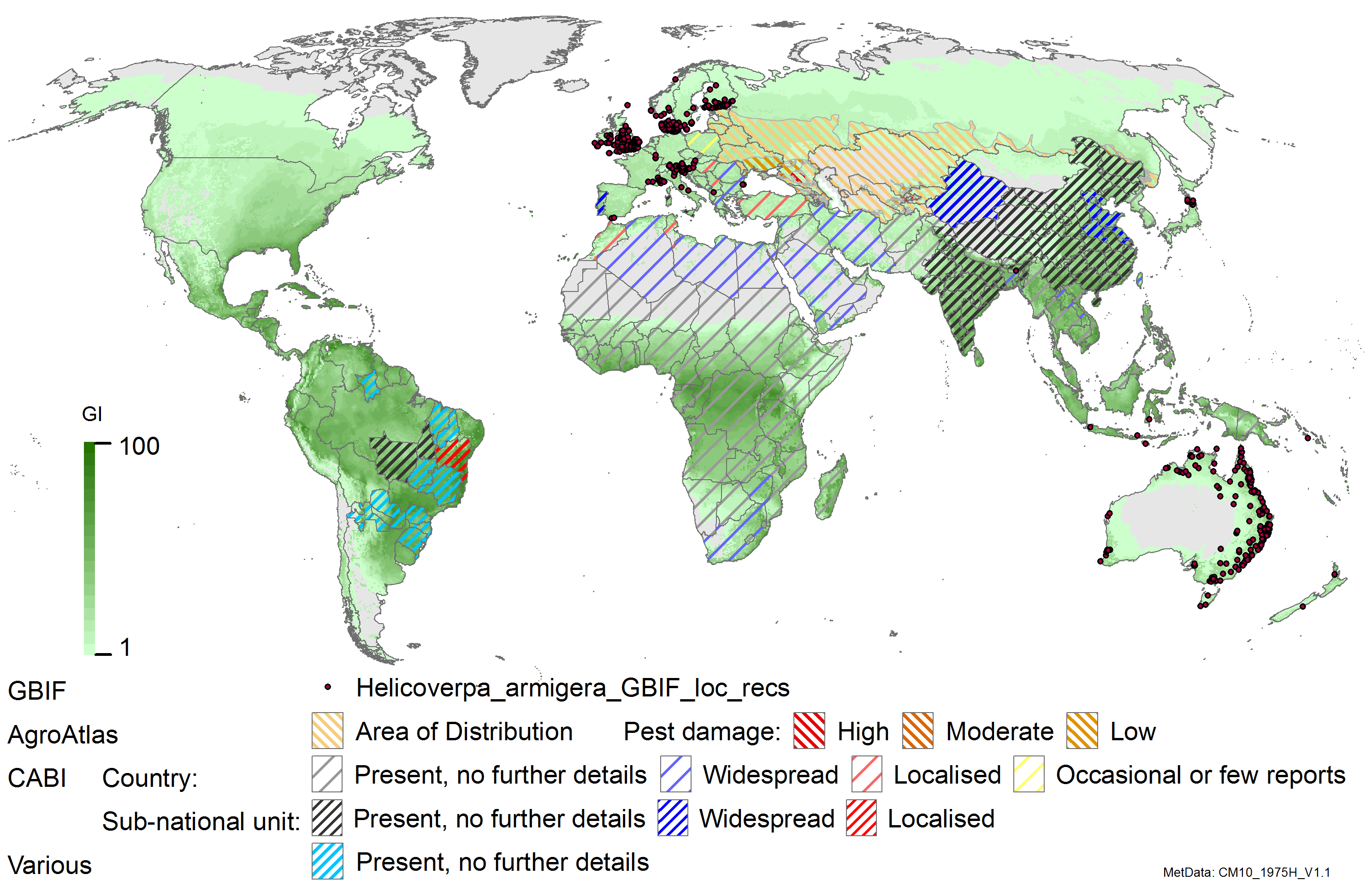


B

A

**Figure B.** Global climate suitability for *Helicoverpa armigera* modelled using CLIMEX for A) Establishment, and B) Population growth in relation to its known distribution. Point locations and shaded areas indicate the goodness of fit of the model.

**Table A. CLIMEX Parameter Sensitivity for *Helicoverpa armigera*.**

| **Parameter** | **Mnemonic** | **Parameter Range** | | | **Change in State Variable** | | | | | | | | | |
| --- | --- | --- | --- | --- | --- | --- | --- | --- | --- | --- | --- | --- | --- | --- |
|  |  | **Low** | **Default** | **High** | **Range** | **Core Dist’n** | **EI** | **GI_A_** | **CS** | **DS** | **HS** | **WS** | **MI** | **TI** |
| Diapause Termination Temperature | DPT1 | 9 | 10 | 11 | 2.99 | 1.08 | 2.7 | 2.8 | 0.2 | 0.6 | 0.0 | 0.0 | 0.00 | 0.0 |
| Limiting low moisture | SM0 | 0 | 0.1 | 0.2 | 2.38 | 0.00 | 2.7 | 2.7 | 0.0 | 0.0 | 0.0 | 0.0 | 14.77 | 0.0 |
| Diapause Induction Daylength | DPD0 | 10.5 | 11 | 11.5 | 0.12 | 1.03 | 1.0 | 1.0 | 0.4 | 1.2 | 0.0 | 0.0 | 0.00 | 0.0 |
| Limiting high temperature | DV3 | 36 | 37 | 38 | 0.09 | 0.00 | 0.7 | 0.7 | 0.0 | 0.0 | 0.0 | 0.0 | 0.00 | 2.0 |
| Lower optimal moisture | SM1 | 0.6 | 0.7 | 0.8 | 0.08 | 0.00 | 2.2 | 2.2 | 0.0 | 0.0 | 0.0 | 0.0 | 9.47 | 0.0 |
| Dry Stress Threshold | SMDS | 0 | 0.1 | 0.2 | 0.04 | 5.00 | 0.6 | 0.0 | 0.0 | 5.9 | 0.0 | 0.0 | 0.00 | 0.0 |
| Limiting low temperature | DV0 | 10 | 11 | 12 | 0.04 | 0.00 | 0.5 | 0.4 | 0.0 | 0.0 | 0.0 | 0.0 | 0.00 | 1.7 |
| Upper optimal temperature | DV2 | 30 | 31 | 32 | 0.04 | 0.00 | 1.9 | 1.9 | 0.0 | 0.0 | 0.0 | 0.0 | 0.00 | 3.8 |
| Degree-days per Generation | PDD | 380 | 475 | 570 | 0.03 | 0.00 | 0.2 | 0.0 | 0.0 | 0.0 | 0.0 | 0.0 | 0.00 | 0.0 |
| Lower optimal temperature | DV1 | 19 | 20 | 21 | 0.01 | 0.00 | 1.1 | 1.1 | 0.0 | 0.0 | 0.0 | 0.0 | 0.00 | 3.3 |
| Wet Stress Threshold | SMWS | 1.9 | 2 | 2.1 | 0.01 | 0.25 | 0.3 | 0.0 | 0.0 | 0.0 | 0.0 | 1.4 | 0.00 | 0.0 |
| Diapause Induction Temperature | DPT0 | 9 | 10 | 11 | 0.00 | 0.01 | 0.3 | 0.3 | 0.0 | 1.9 | 0.0 | 0.0 | 0.00 | 0.0 |
| Heat Stress Temperature Threshold | TTHS | 36 | 37 | 38 | 0.00 | 0.14 | 0.1 | 0.0 | 0.0 | 0.0 | 2.0 | 0.0 | 0.00 | 0.0 |
| Dry Stress Rate | HDS | -0.0048 | -0.004 | -0.0032 | 0.00 | 0.32 | 0.1 | 0.0 | 0.0 | 0.7 | 0.0 | 0.0 | 0.00 | 0.0 |
| Wet Stress Rate | HWS | 0.004 | 0.005 | 0.006 | 0.00 | 0.02 | 0.1 | 0.0 | 0.0 | 0.0 | 0.0 | 0.4 | 0.00 | 0.0 |
| Limiting high moisture | SM3 | 1.9 | 2 | 2.1 | 0.00 | 0.00 | 1.4 | 1.4 | 0.0 | 0.0 | 0.0 | 0.0 | 2.15 | 0.0 |
| Heat Stress Temperature Rate | THHS | 0.0008 | 0.001 | 0.0012 | 0.00 | 0.01 | 0.0 | 0.0 | 0.0 | 0.0 | 0.7 | 0.0 | 0.00 | 0.0 |
| Cold Stress Degree-day Rate | DHCS | -0.0006 | -0.0005 | -0.0004 | 0.00 | 0.03 | 0.0 | 0.0 | 0.2 | 0.0 | 0.0 | 0.0 | 0.00 | 0.0 |
| Cold Stress Degree-day Threshold | DTCS | 4 | 5 | 6 | 0.00 | 0.07 | 0.0 | 0.0 | 0.7 | 0.0 | 0.0 | 0.0 | 0.00 | 0.0 |
| Upper optimal moisture | SM2 | 0.9 | 1 | 1.1 | 0.00 | 0.00 | 1.8 | 1.8 | 0.0 | 0.0 | 0.0 | 0.0 | 6.02 | 0.0 |

**Table B. Frequency of interceptions of *Helicoverpa armigera* in the United States by inspected host.**

|  | **CUT FLOWER** | **CUTTING** | **FRUIT** | **LEAF** | **PLANT** | **SEED** | **STEM** | **WOOD PRODUCT** | **(blank)** | **Grand Total** | **Relative Frequency** |
| --- | --- | --- | --- | --- | --- | --- | --- | --- | --- | --- | --- |
| *Bupleurum* sp. | 63 |  |  | 2 |  |  |  |  |  | 65 | 0.063539 |
| *Ornithogalum* sp. | 53 |  |  |  |  |  |  |  | 2 | 55 | 0.053763 |
| *Leucospermum* sp. | 50 |  | 1 |  |  |  |  |  | 2 | 53 | 0.051808 |
| *Veronica* sp. | 45 |  |  |  |  |  |  |  | 3 | 48 | 0.046921 |
| *Capsicum* sp. |  |  | 22 |  |  |  |  |  | 4 | 26 | 0.025415 |
| *Eryngium* sp. | 24 |  |  | 1 |  |  |  |  | 1 | 26 | 0.025415 |
| *Tagetes erecta* | 25 |  |  |  |  |  |  |  | 1 | 26 | 0.025415 |
| *Rosmarinus officinalis* |  |  | 3 | 20 | 1 |  |  |  | 1 | 25 | 0.024438 |
| *Ocimum basilicum* | 1 |  |  | 17 |  |  |  |  | 2 | 20 | 0.01955 |
| *Cicer arietinum* |  |  | 11 | 2 |  | 3 |  |  | 3 | 19 | 0.018573 |
| *Gerbera* sp. | 17 |  |  |  |  |  |  |  | 2 | 19 | 0.018573 |
| *Dianthus* sp. | 17 |  |  |  |  |  |  |  | 1 | 18 | 0.017595 |
| *Eustoma* sp. | 18 |  |  |  |  |  |  |  |  | 18 | 0.017595 |
| Plant | 14 |  | 1 | 2 |  | 1 |  |  |  | 18 | 0.017595 |
| *Amaranthus* sp. | 14 |  |  | 1 |  |  |  |  |  | 15 | 0.014663 |
| *Anigozanthos* sp. | 15 |  |  |  |  |  |  |  |  | 15 | 0.014663 |
| *Liatris* sp. | 15 |  |  |  |  |  |  |  |  | 15 | 0.014663 |
| *Lisianthus* sp. | 15 |  |  |  |  |  |  |  |  | 15 | 0.014663 |
| *Anemone* sp. | 14 |  |  |  |  |  |  |  |  | 14 | 0.013685 |
| *Helianthus* sp. | 12 |  |  |  |  |  |  |  | 2 | 14 | 0.013685 |
| *Rosa* sp. | 11 |  |  |  | 1 |  |  |  | 2 | 14 | 0.013685 |
| *Tagetes* sp. | 13 |  |  |  |  |  |  |  | 1 | 14 | 0.013685 |
| *Chrysanthemum* sp. | 13 |  |  |  |  |  |  |  |  | 13 | 0.012708 |
| *Ornithogalum arabicum* | 12 |  |  | 1 |  |  |  |  |  | 13 | 0.012708 |
| *Clematis* sp. | 12 |  |  |  |  |  |  |  |  | 12 | 0.01173 |
| *Allium* sp. | 10 |  |  |  |  |  |  |  |  | 10 | 0.009775 |
| *Bupleurum griffithii* | 10 |  |  |  |  |  |  |  |  | 10 | 0.009775 |
| *Origanum vulgare* |  |  | 1 | 8 |  |  | 1 |  |  | 10 | 0.009775 |
| *Salvia officinalis* |  |  | 1 | 7 |  |  |  |  | 2 | 10 | 0.009775 |
| *Salvia* sp. |  |  |  | 10 |  |  |  |  |  | 10 | 0.009775 |
| *Scabiosa* sp. | 10 |  |  |  |  |  |  |  |  | 10 | 0.009775 |
| *Celosia* sp. | 9 |  |  |  |  |  |  |  |  | 9 | 0.008798 |
| Aircraft |  |  | 1 |  |  |  |  |  | 7 | 8 | 0.00782 |
| *Capsicum annuum* |  |  | 8 |  |  |  |  |  |  | 8 | 0.00782 |
| *Delphinium* sp. | 8 |  |  |  |  |  |  |  |  | 8 | 0.00782 |
| *Mentha* sp. |  |  |  | 4 | 2 |  |  |  | 2 | 8 | 0.00782 |
| *Rosmarinus* sp. |  |  |  | 7 |  |  | 1 |  |  | 8 | 0.00782 |
| *Solanum* sp. |  |  | 8 |  |  |  |  |  |  | 8 | 0.00782 |
| *Abelmoschus esculentus* |  |  | 7 |  |  |  |  |  |  | 7 | 0.006843 |
| *Dendrobium* sp. | 6 |  |  |  | 1 |  |  |  |  | 7 | 0.006843 |
| *Polyanthus* sp. | 7 |  |  |  |  |  |  |  |  | 7 | 0.006843 |
| *Ranunculus* sp. | 7 |  |  |  |  |  |  |  |  | 7 | 0.006843 |
| Bouquet | 5 |  |  | 1 |  |  |  |  |  | 6 | 0.005865 |
| *Chamelaucium* sp. | 6 |  |  |  |  |  |  |  |  | 6 | 0.005865 |
| *Origanum* sp. |  |  |  | 5 |  |  | 1 |  |  | 6 | 0.005865 |
| *Phaseolus vulgaris* |  |  | 6 |  |  |  |  |  |  | 6 | 0.005865 |
| *Solanum melongena* |  |  | 4 |  |  |  |  |  | 2 | 6 | 0.005865 |
| Tiles |  |  |  |  |  |  |  | 3 | 3 | 6 | 0.005865 |
| *Cichorium intybus* |  |  |  | 3 |  |  | 1 |  | 1 | 5 | 0.004888 |
| *Moluccella* sp. | 5 |  |  |  |  |  |  |  |  | 5 | 0.004888 |
| *Ocimum* sp. |  | 1 |  | 4 |  |  |  |  |  | 5 | 0.004888 |
| *Rudbeckia* sp. | 5 |  |  |  |  |  |  |  |  | 5 | 0.004888 |
| *Thymus* sp. |  |  |  | 4 |  |  | 1 |  |  | 5 | 0.004888 |
| *Thymus vulgaris* |  |  |  | 4 |  |  | 1 |  |  | 5 | 0.004888 |
| At Large |  |  |  |  |  |  |  |  | 4 | 4 | 0.00391 |
| *Coriandrum sativum* |  |  |  | 3 |  |  | 1 |  |  | 4 | 0.00391 |
| *Eustoma grandiflorum* | 4 |  |  |  |  |  |  |  |  | 4 | 0.00391 |
| *Gentiana* sp. | 2 |  |  |  |  | 1 | 1 |  |  | 4 | 0.00391 |
| *Grevillea* sp. | 4 |  |  |  |  |  |  |  |  | 4 | 0.00391 |
| *Hypericum* sp. | 4 |  |  |  |  |  |  |  |  | 4 | 0.00391 |
| *Lactuca sativa* |  |  |  | 3 |  |  |  |  | 1 | 4 | 0.00391 |
| *Mentha longifolia* |  |  |  | 4 |  |  |  |  |  | 4 | 0.00391 |
| *Phaseolus* sp. |  |  | 4 |  |  |  |  |  |  | 4 | 0.00391 |
| *Pisum sativum* | 1 |  | 2 |  |  |  |  |  | 1 | 4 | 0.00391 |
| *Protea* sp. | 4 |  |  |  |  |  |  |  |  | 4 | 0.00391 |
| *Zea mays* |  |  | 4 |  |  |  |  |  |  | 4 | 0.00391 |
| *Alstroemeria* sp. | 3 |  |  |  |  |  |  |  |  | 3 | 0.002933 |
| *Astrantia* sp. | 2 |  |  | 1 |  |  |  |  |  | 3 | 0.002933 |
| *Bouvardia* sp. | 3 |  |  |  |  |  |  |  |  | 3 | 0.002933 |
| *Brassica* sp. | 1 |  |  | 1 |  |  |  |  | 1 | 3 | 0.002933 |
| *Carthamus* sp. | 3 |  |  |  |  |  |  |  |  | 3 | 0.002933 |
| *Gladiolus* sp. | 3 |  |  |  |  |  |  |  |  | 3 | 0.002933 |
| *Leucadendron* sp. | 3 |  |  |  |  |  |  |  |  | 3 | 0.002933 |
| *Lippia* sp. |  |  |  | 3 |  |  |  |  |  | 3 | 0.002933 |
| *Lycopersicon esculentum* |  |  | 3 |  |  |  |  |  |  | 3 | 0.002933 |
| *Satureja hortensis* |  |  |  | 3 |  |  |  |  |  | 3 | 0.002933 |
| *Anethum* sp. | 1 |  |  | 1 |  |  |  |  |  | 2 | 0.001955 |
| *Antirrhinum* sp. | 2 |  |  |  |  |  |  |  |  | 2 | 0.001955 |
| *Artemisia dracunculus* |  |  |  | 1 |  |  | 1 |  |  | 2 | 0.001955 |
| *Asclepias* sp. | 2 |  |  |  |  |  |  |  |  | 2 | 0.001955 |
| *Astilbe* sp. | 2 |  |  |  |  |  |  |  |  | 2 | 0.001955 |
| Baggage | 1 |  |  |  |  |  |  |  | 1 | 2 | 0.001955 |
| *Brassica oleracea* |  |  |  |  |  |  |  |  | 2 | 2 | 0.001955 |
| *Brunia* sp. | 2 |  |  |  |  |  |  |  |  | 2 | 0.001955 |
| *Calendula* sp. | 2 |  |  |  |  |  |  |  |  | 2 | 0.001955 |
| *Cestrum* sp. | 2 |  |  |  |  |  |  |  |  | 2 | 0.001955 |
| *Cicer* sp. |  |  | 1 |  |  | 1 |  |  |  | 2 | 0.001955 |
| *Cichorium* sp. |  |  |  | 2 |  |  |  |  |  | 2 | 0.001955 |
| *Citrus* sp. |  |  | 1 |  |  |  |  |  | 1 | 2 | 0.001955 |
| *Coriandrum* sp. |  |  |  | 2 |  |  |  |  |  | 2 | 0.001955 |
| *Cucumis sativus* |  |  | 2 |  |  |  |  |  |  | 2 | 0.001955 |
| *Dahlia* sp. | 2 |  |  |  |  |  |  |  |  | 2 | 0.001955 |
| *Fragaria* sp. |  |  | 2 |  |  |  |  |  |  | 2 | 0.001955 |
| *Lathyrus sativus* |  |  |  |  |  | 2 |  |  |  | 2 | 0.001955 |
| *Lathyrus* sp. | 1 |  | 1 |  |  |  |  |  |  | 2 | 0.001955 |
| *Limonium* sp. | 2 |  |  |  |  |  |  |  |  | 2 | 0.001955 |
| *Oxypetalum* sp. | 2 |  |  |  |  |  |  |  |  | 2 | 0.001955 |
| *Pisum* sp. |  |  | 1 |  |  | 1 |  |  |  | 2 | 0.001955 |
| *Tulipa* sp. | 2 |  |  |  |  |  |  |  |  | 2 | 0.001955 |
| *Achillea* sp. | 1 |  |  |  |  |  |  |  |  | 1 | 0.000978 |
| *Ageratum* sp. | 1 |  |  |  |  |  |  |  |  | 1 | 0.000978 |
| *Allium schoenoprasum* |  |  |  | 1 |  |  |  |  |  | 1 | 0.000978 |
| *Ammi majus* | 1 |  |  |  |  |  |  |  |  | 1 | 0.000978 |
| *Ammi* sp. | 1 |  |  |  |  |  |  |  |  | 1 | 0.000978 |
| *Anemone coronaria* | 1 |  |  |  |  |  |  |  |  | 1 | 0.000978 |
| *Anigozanthos manglesii* | 1 |  |  |  |  |  |  |  |  | 1 | 0.000978 |
| *Artemisia* sp. |  |  |  | 1 |  |  |  |  |  | 1 | 0.000978 |
| *Asclepias tuberosa* | 1 |  |  |  |  |  |  |  |  | 1 | 0.000978 |
| *Asparagus* sp. | 1 |  |  |  |  |  |  |  |  | 1 | 0.000978 |
| *Aster* sp. | 1 |  |  |  |  |  |  |  |  | 1 | 0.000978 |
| Automobile parts |  |  |  |  |  |  |  |  | 1 | 1 | 0.000978 |
| *Bellis* sp. | 1 |  |  |  |  |  |  |  |  | 1 | 0.000978 |
| *Berzelia lanuginosa* | 1 |  |  |  |  |  |  |  |  | 1 | 0.000978 |
| *Brassica oleracea var. acephala* |  |  |  | 1 |  |  |  |  |  | 1 | 0.000978 |
| *Brassica rapa* |  |  |  | 1 |  |  |  |  |  | 1 | 0.000978 |
| *Cajanus cajan* |  |  | 1 |  |  |  |  |  |  | 1 | 0.000978 |
| *Capsicum pubescens* |  |  | 1 |  |  |  |  |  |  | 1 | 0.000978 |
| *Capsicum sinense* |  |  | 1 |  |  |  |  |  |  | 1 | 0.000978 |
| Cargo | 1 |  |  |  |  |  |  |  |  | 1 | 0.000978 |
| *Carthamus tinctorius* | 1 |  |  |  |  |  |  |  |  | 1 | 0.000978 |
| *Centaurea* sp. | 1 |  |  |  |  |  |  |  |  | 1 | 0.000978 |
| *Cichorium endivia* |  |  | 1 |  |  |  |  |  |  | 1 | 0.000978 |
| *Cosmos* sp. | 1 |  |  |  |  |  |  |  |  | 1 | 0.000978 |
| *Craspedia* sp. | 1 |  |  |  |  |  |  |  |  | 1 | 0.000978 |
| *Crossandra* sp. | 1 |  |  |  |  |  |  |  |  | 1 | 0.000978 |
| *Cucurbita maxima* |  |  | 1 |  |  |  |  |  |  | 1 | 0.000978 |
| *Cucurbita pepo* |  |  | 1 |  |  |  |  |  |  | 1 | 0.000978 |
| *Cucurbita* sp. | 1 |  |  |  |  |  |  |  |  | 1 | 0.000978 |
| *Cucurbitaceae* | 1 |  |  |  |  |  |  |  |  | 1 | 0.000978 |
| *Cynara scolymus* | 1 |  |  |  |  |  |  |  |  | 1 | 0.000978 |
| *Cynara* sp. |  |  |  |  |  |  |  |  | 1 | 1 | 0.000978 |
| *Diospyros kaki* |  |  | 1 |  |  |  |  |  |  | 1 | 0.000978 |
| *Echinops* sp. | 1 |  |  |  |  |  |  |  |  | 1 | 0.000978 |
| *Erica* sp. | 1 |  |  |  |  |  |  |  |  | 1 | 0.000978 |
| *Fabaceae* |  |  | 1 |  |  |  |  |  |  | 1 | 0.000978 |
| *Gardenia* sp. | 1 |  |  |  |  |  |  |  |  | 1 | 0.000978 |
| *Geranium* sp. | 1 |  |  |  |  |  |  |  |  | 1 | 0.000978 |
| *Gomphrena* sp. | 1 |  |  |  |  |  |  |  |  | 1 | 0.000978 |
| *Gypsophila* sp. | 1 |  |  |  |  |  |  |  |  | 1 | 0.000978 |
| *Hibiscus* sp. |  |  | 1 |  |  |  |  |  |  | 1 | 0.000978 |
| *Hydrangea* sp. | 1 |  |  |  |  |  |  |  |  | 1 | 0.000978 |
| *Jasminum sambac* |  |  |  |  |  |  | 1 |  |  | 1 | 0.000978 |
| *Jasminum* sp. | 1 |  |  |  |  |  |  |  |  | 1 | 0.000978 |
| *Lablab purpureus* |  |  |  |  |  | 1 |  |  |  | 1 | 0.000978 |
| *Lactuca* sp. |  |  |  | 1 |  |  |  |  |  | 1 | 0.000978 |
| Leaf litter |  |  |  |  |  |  |  |  | 1 | 1 | 0.000978 |
| *Leonotis* sp. | 1 |  |  |  |  |  |  |  |  | 1 | 0.000978 |
| *Leucospermum cordifolium* | 1 |  |  |  |  |  |  |  |  | 1 | 0.000978 |
| *Liatris spicata* | 1 |  |  |  |  |  |  |  |  | 1 | 0.000978 |
| *Lilium* sp. | 1 |  |  |  |  |  |  |  |  | 1 | 0.000978 |
| *Lippia graveolens* |  |  |  | 1 |  |  |  |  |  | 1 | 0.000978 |
| *Lycopersicon* sp. |  |  | 1 |  |  |  |  |  |  | 1 | 0.000978 |
| *Lysianthus* sp. | 1 |  |  |  |  |  |  |  |  | 1 | 0.000978 |
| *Lysimachia* sp. | 1 |  |  |  |  |  |  |  |  | 1 | 0.000978 |
| *Malus* sp. |  |  | 1 |  |  |  |  |  |  | 1 | 0.000978 |
| *Malvaceae* |  |  |  | 1 |  |  |  |  |  | 1 | 0.000978 |
| Marble Tiles |  |  |  |  |  |  |  |  | 1 | 1 | 0.000978 |
| *Matricaria* sp. | 1 |  |  |  |  |  |  |  |  | 1 | 0.000978 |
| *Minthostachys* sp. |  |  |  | 1 |  |  |  |  |  | 1 | 0.000978 |
| Miscellaneous spp |  |  |  |  |  |  |  |  | 1 | 1 | 0.000978 |
| Mixed bouquet | 1 |  |  |  |  |  |  |  |  | 1 | 0.000978 |
| *Musa* sp. |  |  |  | 1 |  |  |  |  |  | 1 | 0.000978 |
| *Nerium* sp. | 1 |  |  |  |  |  |  |  |  | 1 | 0.000978 |
| *Olea europaea* |  |  | 1 |  |  |  |  |  |  | 1 | 0.000978 |
| *Oleaceae* sp. | 1 |  |  |  |  |  |  |  |  | 1 | 0.000978 |
| *Oncidium* sp. | 1 |  |  |  |  |  |  |  |  | 1 | 0.000978 |
| *Origanum majorana* |  |  |  | 1 |  |  |  |  |  | 1 | 0.000978 |
| *Ornithocephalus* sp. | 1 |  |  |  |  |  |  |  |  | 1 | 0.000978 |
| *Oxalis tuberosa* |  |  |  | 1 |  |  |  |  |  | 1 | 0.000978 |
| *Phaseolus coccineus* |  |  | 1 |  |  |  |  |  |  | 1 | 0.000978 |
| *Phaseolus lunatus* |  |  |  |  |  | 1 |  |  |  | 1 | 0.000978 |
| *Physostegia* sp. | 1 |  |  |  |  |  |  |  |  | 1 | 0.000978 |
| *Polianthes* sp. | 1 |  |  |  |  |  |  |  |  | 1 | 0.000978 |
| *Polianthes tuberosa* | 1 |  |  |  |  |  |  |  |  | 1 | 0.000978 |
| *Prunus* sp. |  |  | 1 |  |  |  |  |  |  | 1 | 0.000978 |
| *Rumex* sp. |  |  |  | 1 |  |  |  |  |  | 1 | 0.000978 |
| *Satureja* sp. | 1 |  |  |  |  |  |  |  |  | 1 | 0.000978 |
| *Sedum* sp. | 1 |  |  |  |  |  |  |  |  | 1 | 0.000978 |
| *Serruria florida* | 1 |  |  |  |  |  |  |  |  | 1 | 0.000978 |
| *Setaria italica* | 1 |  |  |  |  |  |  |  |  | 1 | 0.000978 |
| Ship deck |  |  |  |  |  |  |  |  | 1 | 1 | 0.000978 |
| *Solanaceae* |  |  | 1 |  |  |  |  |  |  | 1 | 0.000978 |
| *Solanum aethiopicum* |  |  | 1 |  |  |  |  |  |  | 1 | 0.000978 |
| *Statice* sp. | 1 |  |  |  |  |  |  |  |  | 1 | 0.000978 |
| *Syringa* sp. | 1 |  |  |  |  |  |  |  |  | 1 | 0.000978 |
| *Syringa vulgaris* | 1 |  |  |  |  |  |  |  |  | 1 | 0.000978 |
| *Tetrapleura* sp. |  |  |  |  |  |  |  |  | 1 | 1 | 0.000978 |
| *Thymus citriodorus* |  |  |  | 1 |  |  |  |  |  | 1 | 0.000978 |
| *Trachelium* sp. | 1 |  |  |  |  |  |  |  |  | 1 | 0.000978 |
| *Tuberosa* sp. | 1 |  |  |  |  |  |  |  |  | 1 | 0.000978 |
| Unknown |  |  | 1 |  |  |  |  |  |  | 1 | 0.000978 |
| Unknown Leaves |  |  |  | 1 |  |  |  |  |  | 1 | 0.000978 |
| *Vernonia amygdalina* |  |  |  | 1 |  |  |  |  |  | 1 | 0.000978 |
| *Veronica longifolia* | 1 |  |  |  |  |  |  |  |  | 1 | 0.000978 |
| *Viburnum opulus* | 1 |  |  |  |  |  |  |  |  | 1 | 0.000978 |
| *Viburnum* sp. | 1 |  |  |  |  |  |  |  |  | 1 | 0.000978 |
| *Vigna* sp. |  |  | 1 |  |  |  |  |  |  | 1 | 0.000978 |
| **Grand Total** | 676 | 1 | 113 | 142 | 5 | 11 | 10 | 3 | 62 | 1023 | 1 |
